# Youth Engagement, Positive Interethnic Contact, and ‘Associational Bridges’: A Quasi-Experimental Investigation of a UK National Youth Engagement Scheme

**DOI:** 10.1007/s10964-019-01042-x

**Published:** 2019-06-07

**Authors:** James Laurence

**Affiliations:** 1 Cathie Marsh Institute for Social Research, University of Manchester, Oxford Road, Manchester, M13 9PL UK; 2 Wissenschaftszentrum Berlin für Sozialforschung, WZB Berlin Social Science Center, Reichpietschufer 50, 10785 Berlin, Germany

**Keywords:** Social and civic engagement, Inter-group contact, Youth and adolescence, Communities and segregation, Social cohesion, Quasi-experimental

## Abstract

Affective interethnic ties are highly effective for improving out-group attitudes, especially during adolescence. Yet, young people face several obstacles to developing such ties in society. One potential means of fostering greater interethnic connectivity is through youth engagement: that is, formal, organized social participation in groups, clubs, or activities. However, little is still known about its effectiveness; especially for overcoming structural obstacles to contact in society, such as residential segregation. This study has two aims: to robustly examine whether youth engagement can build positive interethnic contact among young people; and, to perform the first systematic test of whether sites of engagement can act as “Associational Bridges”, providing greater opportunities for contact among youth from more segregated environments. To pursue these aims, the study exploited a unique data opportunity to perform a quasi-experimental analysis of a large-scale, nationally-implemented youth engagement scheme in the United Kingdom. The study uses pre-test/post-test data on *N* = 1371 participants (*M*_*age*_ = 16.7; 63% Female; 36% non-White) and *N* = 1946 propensity-score matched controls (*M*_*age*_ = 16.7; 62% Female; 36% non-White). Applying a (matched) difference-in-difference approach, the findings demonstrate that participation led to an increase in affective interethnic ties, which were evident at least 4–6 months after involvement had ended. Participation also had a stronger positive impact on youth from more residentially segregated areas: although joining the scheme with fewer interethnic ties, post-participation they reported just as many ties as their peers from less segregated areas. However, participation had no difference in effects for youth from more or less ethnically diverse areas. Youth engagement may thus be an effective tool for fostering positive contact; particularly among those from more segregated environments. However, attention should be paid to the design and recruitment practices of engagement programs to understand which structural barriers to contact in society they can overcome.

## Introduction

Against a backdrop of polarizing attitudes towards ethnic diversity, the resurgence of far-right groups, and growing support for anti-immigrant parties, the question of how societies maintain positive interethnic relations is of increasing importance (Lambert et al. 2017). This question is especially acute for young people. Adolescence is a key period in the formation of attitudes towards ethnic difference (Kinder and Sears 1981). Concern has also emerged surrounding young people’s susceptibility to extremist messages (e.g. Coppock and McGovern 2014), especially among the socially excluded (Pretus et al. 2018). Furthermore, some evidence suggests younger age-groups may be more likely to be both perpetrators (Brå 2009), as well as victims (Home Office 2013), of hate crimes. With societies set to become increasingly ethnically diverse, it is critical to understand how youth interethnic relations can be strengthened.

One means, frequently advocated across both academic and governmental spheres, is through fostering positive interethnic contact among young people (Pettigrew and Tropp 2006). Yet, there are often-overlooked obstacles to realizing the ameliorative role positive contact can play in society (Dixon et al. 2005). Not all young people have opportunities to mix with different ethnic groups; especially where structural barriers, such as residential segregation (Laurence et al. 2019b), and school segregation (Burgess et al. 2005), inhibit intergroup mixing. Even when opportunities do exist for mixing, for example in diverse schools, it does not necessarily occur (Al Ramiah et al. 2015). Furthermore, when mixing does occur, it may not be of the optimal kind to foster positive contact (Dixon et al. 2005). In fact, when such contact is experienced negatively, it can actually increase prejudice, as observed in diverse schools (Bekhuis et al. 2013), and neighborhoods (Laurence et al. 2017).

In light of these obstacles, youth engagement (that is, formal, organized social participation in a group, club, or activity) could act as a key catalyst for building positive interethnic contact among young people; in particular, for fostering the development of affective interethnic ties, highly effective for prejudice-reduction (Watkins et al. 2007; Knifsend and Juvonen 2017). Not only might youth engagement generate conditions more amenable to fostering optimal intergroup contact (including co-operative, equal-status, common goal-orientated interactions), but when youth engagement brings together young people from different neighborhoods and schools, sites of engagement may act as “Associational Bridges”, helping to overcome structural barriers to intergroup mixing, such as residential segregation. However, while research has begun exploring these questions, shortcomings exist within the current literature. Much of this work relies on cross-sectional data, which limits understanding of the causality of the relationships. Quasi-experimental tests in the field tend to be small-scale, restricting the generalizability of findings. Current work largely focuses on contact during the period of engagement, which prevents insights into whether any gains in contact are maintained *after* participation has ended. Lastly, no systematic tests have explored whether youth engagement is effective for overcoming structural barriers to positive contact, such as segregation.

This study aimed to address these gaps in the current literature to investigate: whether discrete periods of participation through a nationally-implemented youth engagement scheme led to the development of affective interethnic ties (a particularly positive form of contact); whether any gains in interethnic ties were maintained after involvement in the scheme ended; and how far youth engagement was able to overcome structural barriers to mixing in society (in particular, residential segregation). To pursue these aims the study took advantage of a unique data opportunity in the form of a large-scale youth engagement scheme in the United Kingdom: the National Citizen Service. This nationally-implemented program brings together young people to undertake a series of civic and social activities over 3–4 weeks (National Audit Office 2017). All young people aged 16–17 (and some aged 15) are eligible to participate in the scheme, which currently involves high levels of up-take e.g., in 2018, one in six of all those eligible took part (National Citizen Service Trust 2018). Studying the National Citizen Service provides important opportunities to overcome methodological shortcomings in the literature. By applying a quasi-experimental, real-world intervention approach, using pre-test/post-test survey data on a large sample of participants and (matched) controls, drawn from multiple participation-sites across the country, among a more representative sample of young people, the following study could perform a highly robust test of how youth engagement affects positive interethnic contact among young people.

### Young People, Interethnic Contact and its Limitations

Adolescence is a key period of attitude-formation towards ethnic difference (Dunham et al. 2011), after which such attitudes appear to increasingly stabilize over the life course (Henry and Sears 2009). On one hand, during adolescence, peer-group bonding tends to become stronger and ethnic in-/out-group identities and attitudes are heightened, which can weaken intergroup cohesion (Brown 2004). However, adolescence is also when social context (e.g., peers, parents) becomes increasingly important for one’s intergroup attitudes, via processes such as peer-socialization (Hjerm et al. 2018). This makes adolescence a key period during which interethnic contact may be particularly effective for improving intergroup attitudes (Karcher and Fischer 2004).

The contact hypothesis stipulates that social contact between ethnic groups (interethnic interactions) can reduce prejudice (Allport 1954). However, the efficacy of contact for prejudice-reduction depends on several conditions; in particular, the contact should involve equal status encounters between groups, working co-operatively in the pursuit of common goals, in an environment where mixing is sanctioned by authority (such as institutional support or norms) (Pettigrew and Tropp 2006). Pettigrew (1998) stipulated that contact should also involve “acquaintanceship potential”, to facilitate the forming of affective interethnic ties, which are observed to be particularly effective for reducing prejudice. This includes the additional condition of close, sustained interaction, which facilitates processes of self-disclosure and other friendship-developing mechanisms (Pettigrew 1998). When these conditions are met, contact is much more likely to be experienced positively—especially when leading to affective interethnic ties (a particularly positive form of contact)—forming a key means of augmenting youth intergroup relations (Al Ramiah and Hewstone 2012).

While positive contact is effective at improving attitudes towards out-groups, studies suggest it could also have negative side-effects on longer-term social change towards interethnic equality in society. In particular, it has been posited that positive interethnic contact among more disadvantaged-, minority-groups can reduce their support for collective action towards reducing structural inequalities (Cakal et al. 2011; Tausch et al. 2015). This can emerge where forming out-group ties with the advantaged-, majority-group leads to reduced identification with one’s own, disadvantaged-group and increases positive attitudes towards the advantaged group (Tausch et al. 2015), or results in positively biased expectations of equal treatment, and reduced awareness of how disadvantaged one’s own group is (Saguy et al. 2009). All these attitudes can demotivate minority groups’ support for collective action.

While attention should therefore be paid to these potential negative side-effects, positive interethnic contact remains a key means of strengthening intergroup relations among young people. However, there are often overlooked obstacles to realizing the benefits of such positive contact in society.

The first obstacle is that not all young people have opportunities for intergroup contact; particularly where residential segregation inhibits opportunities for mixing in communities (Laurence et al. 2019b). For example, when segregation restricts neighborhood contact, adolescent outgroup bias appears to increase more sharply over time (Merrilees et al. 2018). Residential segregation can also delimit opportunities for contact through more segregated schools, emerging from more homogeneous catchment areas (Burgess et al. 2005). The second obstacle is that even in diverse environments where opportunities for mixing exist, contact does not necessarily follow (Moody 2001). Ethnic homophily is evident in youth social networks (Brown and Klute 2003), which tends to increase during adolescence as ethnic identities become more salient (Brown 2004) and peer-group norms create obstacles to cross-group mixing (Hamm et al. 2005). Studies demonstrate that even in diverse environments in-group contact remains the norm (Maoz 2002). This is starkly realized in some diverse schools where seating patterns in dining halls remain highly segregated (Al Ramiah et al. 2015). The third obstacle is that even when interethnic contact does occur the encounters may not be of sufficient quality to generate positive contact (Dixon et al. 2005). The contact occurring across neighborhoods can be less optimal and superficial (Merrilees et al. 2018). Schools tend to be individualistic-, not co-operative-, orientated environments, which may impede the development of optimal contact (Watkins et al. 2007), while status hierarchies in schools, reinforced by peer-group dynamics, can also inhibit equal-status contact (Hamm et al. 2005). At worse, the contact that does occur in diverse contexts may even be negative, such as interethnic bullying or discrimination observed in school contexts (Bekhuis et al. 2013), or tensions observed in neighborhood contexts (Laurence et al. 2017; Laurence et al. 2019a).

### Youth Engagement and Interethnic Contact

In light of these obstacles, youth engagement could offer a key site for increasing positive interethnic contact. Youth engagement entails formal social participation in an organized club, group or activity, involving some form of co-operative behaviors, undertaken with peers, out of the home (Dworkin et al. 2003). This engagement may occur within schools (e.g. extracurricular activities), or outside of schools, such as uniformed-groups, community groups, or sports clubs. Much like adult associational activity, youth engagement is believed to provide “young people the opportunity to interact and connect with a group of individuals in the pursuit of common goals” (Smith 1999: 555).

The nature of youth engagement could make it effective for overcoming the aforementioned obstacles to positive contact (Knifsend and Juvonen 2017). Engagement activities may be more conducive to optimal social contact (Watkins et al. 2007), nominally involving sustained, co-operative interaction with peers, in the pursuit of common goals (Dworkin et al. 2003). Contact at engagement sites is also more likely to be equal-status (particularly when it occurs in non-school settings, away from school peer-group pressures), while sites of engagement also seek to create norms sanctioning positive interactions (Watkins et al. 2007). These conditions, coupled with the close, sustained interaction often experienced during participation, means youth engagement also likely has a strong potential to lead to affective-tie formation (Pettigrew 1998). Accordingly, the following model can be proposed: youth engagement increases opportunities for interethnic contact; the nature of this contact is likely to be more optimal; this, in turn, will mean the contact experienced is more positive and has a greater potential to lead to affective interethnic ties, which themselves are more likely to be sustained *after* participation has ended.

Critically, youth engagement may also create greater opportunities for positive interethnic mixing among young people who have fewer opportunities in their everyday lives; particularly among those living in more segregated communities (Cairns and McKeown 2012). When youth engagement activities are organized outside of schools, and across neighborhoods, they may be able to act as “Associational Bridges”, providing opportunities to build greater contact among youth from more segregated environments.

At the same time, there may be limits to youth engagement. Contact through engagement may remain sub-optimal, and homophily may still prevail (Conner and Erickson 2017). Furthermore, any positive contact may not last beyond the end of involvement. This may be especially pertinent for young people from segregated areas who, after engaging, return to their more homogeneous social environments, making sustaining any gains in interethnic contact more difficult (McKeown and Taylor 2017). How then does the current research speak to these questions?

### Current Evidence on the Role of Youth Engagement for Interethnic Contact

Research in this area often focuses on youth engagement within schools. Single-site school studies (Aboud et al. 2015), or studies using US state- (Knifsend and Juvonen 2017) or nationally-representative (Moody 2001) surveys of pupils, find young people involved in extra-curricular activities generally report more positive contact than their non-engaged peers. These studies also suggest as the diversity of one’s co-participants increases the amount of positive contact young people experience also increases, measured either directly, using self-reported indicators of co-participant diversity (Knifsend and Juvonen 2017), or proxied using statistical measures of school diversity (Jones et al. 2016). Although, at the same time, ethnic segregation within groups/activities can still occur, even in diverse schools (Moody 2001).

While this research is suggestive that youth engagement can boost contact, it has several limitations. In focusing on *within*-school engagement, opportunities for contact are dependent on the ethnic composition of schools. This provides less insight into how youth engagement can foster contact among individuals from segregated environments; although, studies in Northern Ireland show how Catholics and Protestants involved in *non*-school, cross-community programs can report greater contact (Cairns and McKeown 2012). A more critical limitation of this work however is its reliance on cross-sectional data. Cross-sectional associations between participation and contact may not be causal but driven by other factors e.g., certain types of people (more prosocial, open to experience, extroverted) may be more likely to both participate (Binder and Freytag 2013) but also have more outgroup contact (Vezzali et al. 2018). Similarly, cross-sectional approaches limit confidence in those findings showing that participating in more diverse schools (with a more diverse set of co-participants) necessarily causes bigger gains in intergroup contact, relative to those participating in less diverse environments. Does the engagement itself drive this additional contact? Or, do young people participating in more diverse schools simply have more positive contact *prior* to participating (by virtue of already being in a diverse environment)?

Considering these limitations, quasi-experimental (pre-test/post-test) investigations of *non*-school youth engagement schemes provide a robust approach to testing the participation-contact link. Several such studies demonstrate corroborating support for the effect of participation on contact. A single-site US study of a week-long program of team-building and community-service showed that, post-participation, young people reported increased optimal contact (Seaman et al. 2010). A larger scale, Malaysian study of a 3-month engagement program showed that some participants experienced greater optimal contact relative to a control-group (Al Ramiah and Hewstone 2012). There is also evidence that non-school engagement schemes may be effective at fostering positive contact for youth from more segregated communities. A small study of *n* = 56 Protestants and Catholics, recruited from segregated communities, showed that after a 3-day workshop, pre-participation segregated seating patterns had completely disappeared at the conclusion of the program (McKeown et al. 2012). However, other work tempers these findings.

One of the largest studies (*n* = 4210 participants/controls) analysed a national, 10-month engagement program (AmeriCorps) in the US, focused on community-service activities. After the scheme ended, participants were less likely to be involved in and endorse interethnic contact compared to a control-group (Frumkin et al. 2009). Important insights can also be found among contact-intervention schemes, which aim to mix young people from different backgrounds through various group-activities. In a one-year study of 46 separate Arab-Jewish mixing schemes in Israel, Maoz (2002) found that in 15% of cases contact was “very low or entirely absent”. Therefore, even when the explicit aim of engagement is to foster positive mixing it does not necessarily occur (although this may be higher in conflict-regions).

On balance, studies reveal promising evidence that participation can boost contact. However, the cross-sectional nature of much of this work makes causal claims difficult, and while quasi-experimental studies provide some supportive evidence, these are often conducted at one or two sites, limiting how far findings can be generalized outside of their studies. In contrast to these smaller-scale studies, the available larger-scale, quasi-experimental studies tend to find less supportive evidence. There are also no systematic tests of whether participation can overcome structural barriers to contact such as residential segregation. In addition, studies rarely examine whether any positive contact is sustained after participation ends; most studies measure contact either during, or directly after, engagement.

### Background of the National Citizen Service Scheme: Design and Key Features

This study aims to address these outlined gaps and limitations within the current evidence-base to significantly advance the field’s understanding of how youth engagement affects positive interethnic contact. To do so, it will explore the impact of engagement through an analysis of a nationally-implemented youth engagement scheme in the United Kingdom: the National Citizen Service. This section outlines the design and key features of the National Citizen Service program, and will highlight their relevance for the study’s aims of exploring how youth engagement affects interethnic contact.

National Citizen Service is a nationally-implemented youth engagement scheme, involving 15–17 year olds participating in small teams of 12–15 people over a 3–4 week period (National Audit Office 2017). The summer cohort of the scheme comprises three-phases of activities. Phase 1 involves outward-bound team-activities, such as raft building and outdoor pursuits. Phase 2 involves a residential for participants to experience independent living and build life-skills in personal/civic competency. This involves staying away from home (often in University accommodation) and learning new skills, such as communication-skills or setting up social action projects, with practitioners and trainers. In phase 3, participants return home and commence 60-hours commitment to the design and implementation of a social action project; for example, building a communal garden (National Audit Office 2017).

Eligibility for participation is determined solely by age. To participate individuals have to be 16- or 17 years old; only 15-year olds who turn 16 by August 31^st^ of the participation-year are also eligible. Parents/guardians also pay £50 per participant. However, aid is in place to ensure financial-status is not a determinant of participation, and where families experience financial constraints the costs are reduced or waived entirely (National Audit Office 2017). Importantly, the composition of participants is broadly reflective of the eligible youth population. Scheme administrative data suggests that across key socio-demographics participants largely reflect the composition of the eligible youth population, but with some overrepresentation among females (59% on the scheme versus 49% in society), ethnic minorities (32% versus 20%), young people eligible for free school meals (17% versus 8%), and those from the top quantile of community deprivation (13% versus 11%) (National Audit Office 2017).

Several key features of the scheme’s design have direct implications for the study’s expectations of how participating on National Citizen Service might impact young people’s interethnic ties. Firstly, the scheme’s activities fulfill many of the optimal conditions outlined for positive interethnic contact, including structured (task-/skill-related), co-operative activities, within the structural parameters of a defined group, in the pursuit of common goals. National Citizen Service also explicitly aims to foster a positive environment between young people, providing authority sanctioning cross-group mixing (National Audit Office 2017). Furthermore, the close, sustained contact over 3–4 weeks has strong “acquaintanceship potential”. Therefore, in theory, the structure of the scheme should lead to greater positive contact; particularly, the formation of affective interethnic ties.

Secondly, another key feature of the scheme is that it does not occur within schools. Instead, the scheme is designed so that young people engage with other participants drawn from across the same wider community (Local Authority) in which they live. Local Authorities are local government administrative areas in the United Kingdom (mean = 230,000 people). Critically, the scheme aims to ensure that the ethnic mix of participants within any given Local Authority reflects the ethnic composition of the broader Local Authority youth population; for example, if 20% of 15–17 year-olds in a Local Authority are Asian the aim is to ensure 20% of participants within the Local Authority are Asian. Providers of the scheme are incentivized to accomplish this through rebated project costs if the target is achieved. In 2015, 88% of Local Authorities had a profile of participants that matched the ethnic profile of the youth Local Authority population (National Audit Office 2017). Where parity was not achieved this was largely a consequence of over-representation of minority groups.

This *within*-Local Authority design may have implications for how participation impacts young people coming from Local Authorities with different levels of: (a) segregation; and (b) ethnic diversity. Regarding residential segregation, in more segregated Local Authorities young people are more likely to live in ethnically homogeneous neighborhoods and attend more homogeneous schools, restricting contact opportunities (Burgess et al. 2005; Laurence et al. 2019b). However, as the scheme is designed to make the ethnic composition of one’s co-participants representative of their wider Local Authority, the scheme will bring together young people from different neighborhoods and schools from *across the Local Authority* to achieve this. Therefore, participants in segregated areas should experience increased opportunities to mix with ethnic outgroup members through participation that they normally have little contact with in their Local Authority. This should, in turn, result in a stronger impact of participation on their interethnic ties, relative to their peers in integrated areas who likely already have frequent opportunities for contact in their everyday lives.

Regarding levels of ethnic diversity in a young person’s Local Authority, if the design of the scheme means one’s co-participants are more likely to reflect the ethnic composition of their wider Local Authority then young people from more diverse areas should encounter more ethnic out-group co-participants during their involvement. This should result in greater opportunities for positive interethnic contact among participants from more diverse Local Authorities, leading to a stronger impact on their levels of interethnic ties.

In sum, this study posits that the nature of the scheme’s activities should facilitate an increase in participants’ affective interethnic ties. However, its *within*-Local Authority design could result in different impacts for those from more or less segregated areas and more or less diverse areas.

## Current Study

The present study has two key aims: to explore how involvement in youth engagement activities impacts the development of interethnic ties; and to test whether sites of youth engagement can act as “Associational Bridges”, providing greater opportunities for forming interethnic ties among youth from more segregated environments. These aims are pursued through an analysis of the National Citizen Service scheme, which provides important opportunities to overcome limitations within the current literature. Firstly, this study undertakes a quasi-experimental test of a large-scale, nationally-implemented engagement scheme, using participants from multiple participation-sites across the country, on a more representative sample of young people. Secondly, it will explore whether any improvements in positive contact are sustained at least 4–6 months *after* the participation has ended. Thirdly, studying the National Citizen Service provides the opportunity to perform the first systematic test into whether youth engagement exerts differential impacts on interethnic tie formation between participants coming from more or less segregated areas.

Drawing on the current literature, and based on the outlined design and key features of the engagement scheme, hypotheses can be developed on how participation is predicted to impact interethnic ties. Firstly, given the scheme’s activities fulfill many of the conditions for optimal intergroup contact with high “acquaintanceship potential”, participation on the National Citizen Service is predicted to increase participants’ affective interethnic ties (Hypothesis 1). Secondly, as outlined, the scheme is designed so that participants engage with others from the same Local Authority, and it aims to make the ethnic composition of participants in any given Local Authority representative of the youth Local Authority population as a whole. Accordingly, participants in more segregated areas, who normally have fewer day-to-day opportunities for interethnic contact, are predicted to experience greater gains in interethnic ties through participation (Hypothesis 2). In addition, this within-Local Authority design should also mean that participants living in more diverse areas are predicted to have more opportunities for interethnic contact during their engagement, given their co-participants will likely be more diverse. Accordingly, participants in more diverse Local Authorities are expected to experience a stronger positive impact on their interethnic ties (Hypothesis 3).

## Methods

### Study Design

Pre-participation and post-participation data were collected on a sample of participants who took part during summer, 2015, and a control-group of young people. As the scheme is available to all eligible young people, randomized assignment was not possible, constituting a non-equivalent control-group design. Accordingly, this study undertakes a difference-in-difference (DiD) approach, which compares the pre-test/post-test changes in positive contact between the participant/control-group. Unbiased causal-estimates depend on the assumption that, absent of engagement, participants and controls would have exhibited similar pre-test/post-test changes in the outcome. Two steps are taken to strengthen this assumption.

Firstly, the control-group is composed of young people who engaged in the recruitment process for the National Citizen Service but did not finally participate i.e. an expression of interest group (e.g., McAdam 1986). These young people attended a recruitment event, provided their details for further information, but did not go on to participate in summer 2015^1^. This group should be more similar to the National Citizen Service participants than young people in general given their active engagement with the recruitment process. Secondly, to further enhance similarity of the participant-/control-group samples, a propensity-score matching (PSM) exercise in undertaken across an extensive range of pre-participation measures (*see below*). This constitutes a propensity score matched difference-in-difference approach (PSM-DiD).

This quasi-experimental test of a large-scale, real-world intervention helps address methodological limitations among current studies. With a fixed-effects component, DiD-approaches test how participation is related to changes over time in the outcome. This removes the impact of time-invariant unobserved heterogeneity from model estimates, such as personality traits (Bärnighausen et al. 2017). Another potential issue is the presence of time-variant unobserved heterogeneity i.e., that something else changes in participants’ lives driving both decisions to participate and changes in interethnic ties (Fujiwara and Kawachi 2008). However, the quasi-experimental, intervention approach also helps address this, as the key difference between the participant and control samples is that the former undertook a period of engagement and that this engagement occurred just after the pre-test measure of interethnic ties was taken. Similarly, the intervention-approach helps address reverse causality: knowing that the participation occurred *between* the pre-test/post-test measures of interethnic ties provides evidence it is unlikely that the formation of out-group ties drives participation.

### Sample and Data

Data collection was commissioned by the Department for Digital, Culture, Media and Sport (DCMS). Pre-test data on participants was gathered prior to the start of activities. Questionnaires were administered to all participants who took part during a four-week evaluation window during summer 2015^2^. Follow-up surveys were then completed 4–6 months after participation ended. For the control-group, a random sample of individuals was selected from the expression-of-interest pool of non-participants (*see above*). The control-group was surveyed over the same period as participants. A mixed-mode approach was taken, including postal surveys at baseline and both postal and online surveys at follow-up. Response rates to the baseline survey were 85% among participants and 41% among controls. A random sample of *n* = 3985 participants and *n* = 3985 controls was then invited to complete the follow-up survey. Post-participation follow-up response rates for participants and controls were 52% and 51%. The higher baseline response-rate among participants is likely driven by the surveys being part of broader information gathered from participants prior to starting Phase 1 of the scheme. The control-group baseline response rate compares favorably with other large-scale (government) youth surveys (especially given youth remain a relatively harder to reach population when surveyed outside of classrooms), while the follow-up response rates are in line with other two-wave, non-classroom-based youth surveys (Brown et al. 2008; ARK 2009; Jessiman and Drever 2010; Keating et al. 2010; Gireesh et al. 2018).

Non-response and missing within-case data can threaten the external-validity of the analysis. Several baseline factors are associated with post-test survey non-response, including being male, reporting lower generalized trust and exhibiting less frequent civic engagement^3^. Reassuringly, similar drivers of non-response operate among both participants and controls, and baseline positive contact does not predict attrition. Missing within-case data also never exceeds 2% on any one variable. However, extensive testing using weighting and multiple-imputation to address potential bias is also undertaken (see Robustness and Sensitivity section).

### Measures

#### Outcome: affective interethnic ties

The key outcome of interest is whether engagement leads to affective interethnic ties; a key indicator of positive interethnic contact (Lolliot et al. 2015), and a well-established strong predictor of prejudice-reduction (Pettigrew 1998). This follows the proposed model that engagement should trigger increased opportunities for optimal interethnic contact with strong “acquaintanceship potential”, leading to the formation of interethnic ties. Affective interethnic ties are captured using the following question: “Now, think about people you know who you would feel happy getting in touch with to ask for advice or a favor. How many are from a different race or ethnicity to you?” Responses on a 4-point Likert scale included (0) “none of them”, (1) “hardly any of them”, (2) “some of them”, and (3) “many of them”. This question is designed to capture social ties with a strong, affective component, which involve the provision of support, confiding/disclosure and reciprocity (Pettigrew 1998).

#### Area segregation and area ethnic diversity

As discussed, the scheme is designed so that the composition of participants aligns with the composition of the youth population in the wider area (Local Authority) which young people participate in. Therefore, all contextual-level variables are measured at the Local Authority-level. A multi-group measure of segregation is applied to better capture the complexity of residential segregation among groups in an area, using the multi-group entropy index (H Index) (Iceland 2004). Segregation is measured between five ethnic groups: White, Black, Asian, Mixed and Other^4^. The Output-Area (mean *n* = 300 people) forms the lower area unit nested within the Local Authority. The H-Index captures how (un)evenly ethnic groups are distributed across all the Output Areas that compose a Local Authority. Values range from 0 (perfectly integrated) to 1 (perfectly segregated):

$$H = \mathop {\sum}\limits_{i - 1}^n {\left[ {\frac{{t_i\left( {E - E_i} \right)}}{{ET}}} \right]}$$

where *t*_*i*_ refers to the total population of OA *i*. *T* is the Local Authority area population. *n* is the number of OAs. *E*_*i*_ represent OA *i*’s diversity (entropy) and *E* represents Local Authority area diversity (entropy) (see below for calculation of entropy scores).

The H-Index falls into the class of segregation indices tapping the (un)eveness with which ethnic groups are distributed across an area (Massey and Denton 1988). The calculation of such indices are largely unaffected by the size of groups in an area^5^, instead capturing the *relative* distribution of those groups that are present across the area (Laurence et al. 2019b). This results in a greater spread of different levels of segregation across different levels of diversity (the two are correlated at *r* = 0.1). This provides opportunities to test whether, after controlling for the size of groups in an area, the unevenness with which those groups are distributed across it affects the impact of participation.

To measure the level of ethnic diversity in a Local Authority the Entropy Score is used for harmonization with the H Index. The Entropy score captures how far the ethnic composition of an area departs from perfect homogeneity (correlating with other measures of diversity e.g. Simpson’s: *r* = 0.99). A maximum score is when all groups have equal representation in the area:

$$E = \mathop {\sum}\limits_{r = 1}^r {\left( {{\it{{\Pi}}}_r} \right)} \ln \left[ {l/{\it{{\Pi}}}_r} \right]$$

where Π_r_ refers to a particular ethnic group’s proportion of the whole Local Authority population. Logarithmic calculations use the natural log (Iceland 2004).

Complete data on the ethnic composition of the teams in which young people participate is not available to link into the data. However, as discussed, the scheme aims to make the ethnic composition of participants within any given Local Authority representative of the ethnic composition of the youth Local Authority population. Best available estimates put the correlation between participants’ team ethnic diversity and Local Authority ethnic diversity at *r* = 0.76^6^. Local Authority ethnic diversity is therefore also used as a proxy for participants’ opportunities for interethnic contact on the scheme.

### Analytic Approach

#### Propensity score matching

Participants/controls are matched on an extensive set of pre-participation characteristics (see Table 2 in the Appendix for full descriptives). Alongside socio-demographics, matching occurs on key attitudinal/behavioral measures, including: civic and informal helping activities, generalized trust, an index of social confidence^7^, as well as intergroup ties to strengthen claims of strong ignorability. Matching also occurs on Local Authority-level ethnic diversity, segregation, and an index of socio-economic disadvantage (percent in social housing, percent female-headed lone-parent households, percent unemployed), alongside region of residence. Matching on this extensive set of covariates should significantly strengthen the parallel-trends assumption.

To achieve the best balance between participants/controls multiple-matching specifications are run, including restricting to regions of common support, trimming (5% level), and caliper/bandwidth specifications. Three matching specifications return similarly low-levels of bias (*see* Supplementary-Appendix A.1 for a comparison across strategies). This study takes an Epanechnikov kernel-density matching approach with a bandwidth of 0.06, trimmed at the 5% level (see Supplementary-Appendix A.2 for pre-/post-matched sample characteristics and A.3–A.4 for post-matching diagnostics). Kernel-density matching compares each participant with all available control observations, weighting observations according to their distance (Propensity Score) from participant cases. However, consistent findings are returned for all three low-bias strategies (*see* Robustness and Sensitivity section).

#### Modeling

The second stage of a PSM-DiD approach involves regression modeling to estimate the DiD-scores (the impact of participation), incorporating the kernel-density PSM weights. With two-waves of data (pre-test and post-test), and individuals nested within Local Authorities, two-level hierarchical mixed-effects linear regression models with robust standard errors were applied, to account for clustering of the residuals. The DiD-score is estimated via an interaction between a control-/participant-group dummy variable and a pre-test/post-test dummy variable. This represents the fixed-effects component of the models, testing whether participation is associated with changes over time in the outcome at the individual-level. As PSM-approaches can bias variance models also apply bootstrap procedures (1000 reps). To test whether participation exerts a significantly different effect on positive contact depending on the level of segregation or ethnic diversity in a young person’s Local Authority a difference-in-difference-in-difference (DiDiD) approach was taken (*see below*).

## Results

**Fig. 1:**
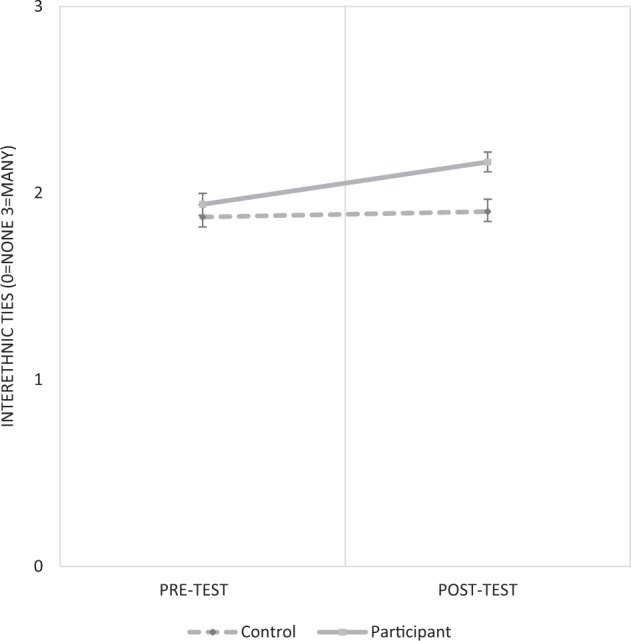
Overall impact of participation on interethnic ties

The first step of the analysis tested the overall impact of participating on young people’s affective interethnic ties (H1). Model 1 (Table 1) shows the DiD-term for the impact of participation is significant and positive. Based on Model 1, predicted pre-test/post-test scores of interethnic ties are plotted for participants and the control-group (Fig. 1). Participants saw a significant increase in interethnic ties (first-difference (FD) score: 0.23 [*CI: 0.18, 0.27*]). Controls exhibited no significant pre-test/post-test difference in ties (FD: 0.03 [*CI:* −0*.03, 0.09*]). This represents a DiD-score of 0.2 [*CI: 0.12, 0.27*]. Young people who participated therefore saw a significant increase in their affective interethnic ties, which were evident at least 4–6 months *after* they completed their participation. However, the size of the increase (0.2 on a 4-point scale), while non-trivial, remains relatively small. These results provide evidence in support of H1.

**Table 1:** Impact of youth engagement on interethnic ties

	Model 1	Model 2	Model 4	Model 5	Model 5
Dependent variable	Interethnic ties	Interethnic ties	Interethnic ties	Interethnic ties	Interethnic ties
Post-test (cf. pre-test)	0.029 (0.032)	0.029 (0.032)	0.064 (0.070)	0.138** (0.051)	0.14 (0.070)
Participant (cf. control-group)	0.068 (0.039)	0.017 (0.036)	0.026 (0.100)	0.184** (0.060)	0.137 (0.098)
Post-test × participant (DiD)	0.197*** (0.037)	0.197*** (0.037)	0.046 (0.076)	0.168** (0.064)	0.046 (0.087)
Area segregation		−0.659*** (0.188)	−0.86* (0.444)	−0.779*** (0.194)	−1.245*** (0.373)
Area ethnic diversity		0.646*** (0.046)	0.653*** (0.047)	0.962*** (0.097)	1.000*** (0.105)
Post-test × area segregation			−0.213 (0.364)		−0.043 (0.345)
Participant × area segregation			−0.058 (0.608)		0.391 (0.530)
Post-test × participant × area segregation (DiDiD)			0.931** (0.348)		0.740* (0.357)
Post-test × area ethnic diversity				−0.198* (0.087)	−0.196* (0.090)
Participant × ethnic diversity				−0.360*** (0.108)	−0.400*** (0.116)
Post-test × participant × area ethnic diversity (DiDiD)				0.059 (0.095)	0.062 (0.097)
Constant	1.872*** (0.034)	1.716*** (0.045)	1.736*** (0.072)	1.593*** (0.052)	1.651*** (0.066)
N (individuals)	3311	3311	3311	3311	3311
N (Local Authorities)	302	302	302	302	302

Kernel-density (Epanechnikov) propensity-score weighted; bootstrapped standard errors in parentheses (1000 reps)

*p < 0.05, **p < 0.01, ***p < 0.001, (two-tailed tests)

The next aim is to test whether participation had different effects on interethnic ties depending on the type of area that young people came from. Firstly, the associations between segregation/diversity and levels of interethnic ties were explored. Model 2 (Table 1) adds Local Authority-level residential segregation and ethnic diversity to the model. Segregation has a significant negative association with interethnic ties while ethnic diversity has a significant positive association. Therefore, where diversity was higher in an area young people reported more ties, while, at the same time, where segregation was higher young people reported fewer ties.

To test whether the effect of participation on a young person’s interethnic ties depended on where they lived a DiDiD-approach is taken, applying a three-way interaction-term between the pre-/post-test identifier, control/participant identifier, and the level of segregation or diversity in a young person’s Local Authority. Model 3 tests whether the impact of participation differed depending on the level of segregation in a young person’s Local Authority. As can be observed, the DiDiD-term is positive and significant, suggesting participants from segregated areas saw a bigger increase in their affective interethnic ties compared to participants from more integrated areas. Model 4 tests whether the impact of participation differed depending on the ethnic diversity of a young person’s Local Authority. As can be observed, the DiDiD-term for ethnic diversity is weakly positive but non-significant; participation thus exerted a similarly sized positive impact among young people from low- and high-diverse areas. Model 5 then tests both DiDiD-terms simultaneously: the findings remain substantively unchanged.

**Fig. 2:**
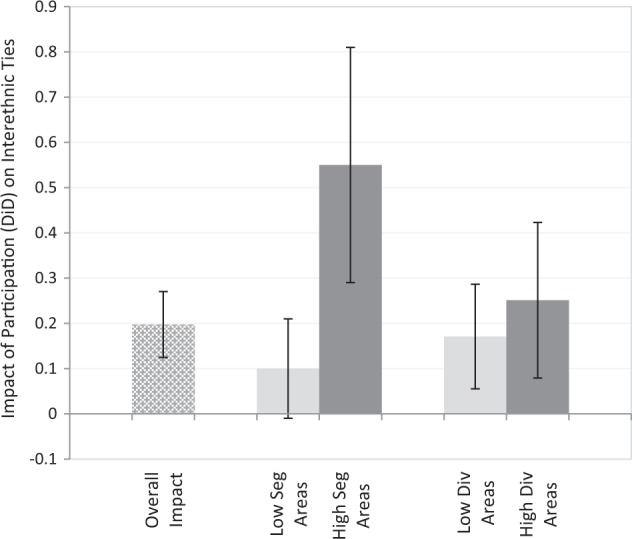
Summary of the impact of participation (DiD-scores) on interethnic ties: among all participants (overall); and among youth from low/high diversity/segregation areas. Notes: Impact of participation scores (DiD) represent the pre-test/post-test change in intergroup ties; *Seg* segregation, *Div* diversity

**Fig. 3:**
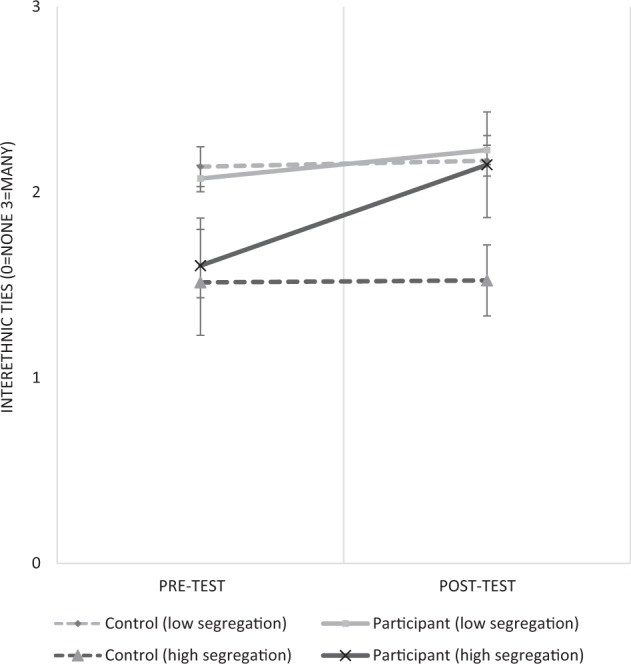
Impact of participation on interethnic ties among youth from low and high segregated areas. Notes: low segregation (H) = 0.05; high segregation (H) = 0.5

The overall, and differential, impacts of participation on interethnic ties are summarized in Fig. 2. Firstly, this demonstrates evidence in support of the second hypothesis (H2): that participation would have a stronger impact on interethnic ties among youth from segregated areas. To examine the implications of this finding in more detail Fig. 3 plots predicted pre-test/post-test scores of interethnic ties for participants/controls from the least (H: 0.056) and most (H: 0.5) segregated Local Authorities in the sample (derived from Model 5). Pre-participation, young people from less segregated Local Authorities joined the scheme with more interethnic ties than their peers from segregated areas. However, they only experienced a small and (marginally) non-significant increase in ties (DiD-score for the impact of participation among integrated youth: 0.1 [*CI: −0.003, 0.22*]), joining and leaving the scheme with the same, relatively high, levels of interethnic ties. Young people from segregated communities joined the scheme with significantly fewer ties. However, they also experienced a much larger increase in interethnic ties from engaging (DiD-score for the impact of participation on segregated youth: 0.5 [*CI: 0.29, 0.8*]). The result is that, post-participation, young people from segregated areas had largely closed the gap in affective interethnic ties with their peers from less segregated areas^8^.

Secondly, Fig. 2 also highlights the lack of evidence in support of H3: that participation would have a stronger impact on interethnic ties among youth from more, compared to less, diverse areas. Instead, both groups reported similar positive impacts. One explanation for this may be that youth in more diverse areas already joined the scheme with relatively high-levels of interethnic ties. As Model 5 (Table 1) shows, Local Authority ethnic diversity has a strong positive association with young people’s interethnic ties. Predicted scores show that, before joining, youth in the most diverse areas reported interethnic ties 1-point higher (on the 4-point scale) than their peers in the least diverse areas^9^. As such, despite having more opportunities to form interethnic ties through participation (given their teams are more diverse), because they already possessed relatively higher levels of ties *before* joining this may limit how many additional ties they gained from taking part. Concurrently, although youth from less diverse areas joined the scheme with fewer interethnic ties, they also likely had fewer opportunities to form ties during participation (given their teams are less diverse), limiting how many additional ties they could gain from taking part. Taken together, these dual-processes, occurring among participants in higher- and lower-diversity areas, could account for why there is no significant difference in the impact of participation between them. This issue is discussed further in the Discussion section.

### Robustness and Sensitivity Testing

#### Tests of internal and external validity

One potential issue with the internal-validity of the study is that, given selection on to the scheme is non-random, young people choosing to participate may already have had increasing rates of interethnic ties *prior* to engaging, which is driving the apparent positive impact of engagement. To gain some purchase on this, one approach is to test whether young people who selected on to the scheme, but who reported that they did not gain anything from their experience, saw no impact of participation on their interethnic ties (*see* Supplementary-Appendix B). The assumption being, participants who did not find the scheme enjoyable are less likely to have developed affective interethnic ties during engagement. If those who did not ‘enjoy’ their involvement experienced no gains in interethnic ties, this might suggest the observed impact of participation is not simply driven by a positive pre-participation contact trend among all those who chose to participate on the scheme; instead, that the participatory-experience itself mattered.

To explore this, a measure of whether participants reported finding the scheme enjoyable is used: “Thinking about your National Citizen Service experience overall…On a scale from 0–10, where 0 is not at all enjoyable and 10 is completely enjoyable, how enjoyable did you find this experience?” Most reported their experience as 10, “completely enjoyable” (41%) and 81% reported 8 or above for how enjoyable it was. Young people are therefore divided into two categories: those who found the scheme less enjoyable (rated 0–7) and more enjoyable (8–10). After splitting participants into groups of more/less enjoyable, kernel-density matching is used to create two matched samples of controls for the more-enjoyable and the less-enjoyable participant groups.

The results show that those participants who found the scheme enjoyable saw a positive impact of participation, relative to the control-group (DiD-score: 0.23 [CI: *0.16, 0.31*]). However, participants who did not find the experience enjoyable saw no significant impact (DiD-score: −0.002 [*CI: −0.13, 0.122*]). Observing that not all participants experienced an increase in interethnic ties, and that the increase appears conditional on participants’ experience of the scheme, suggests the observed effect of participation is less likely to be driven by a different *pre*-participation trend in ties among participants. Furthermore, one’s ‘enjoyment’ of the scheme could potentially be conceived as a proxy for the quality of contact participants had with co-participants during their involvement. Accordingly, these findings could also suggest that contact only tends to generate affective interethnic ties when the contact is positive.

To explore threats to external-validity from survey non-response/missingness, weights and multiple-imputation methods are applied. Reassuringly, tests return substantively similar findings (*see* Supplementary-Appendix C for full discussion and results). Tests also examined the sensitivity of findings to matching/model specifications, and control-group choice, including: running models using alternative (similarly low-bias) PSM-approaches; performing doubly-robust regressions; and testing an alternative control-sample drawn from a nationally-representative data set. These tests returned substantively similar findings (Supplementary-Appendix D).

#### (Inter-)Relationships between community diversity, community segregation and participation

A key take-away from Model 5 is that, holding constant whether participation exerts stronger/weaker effects across different levels of Local Authority diversity, what matters for how participation impacts inter-ethnic ties is how (un)evenly that ethnic diversity is distributed across the Local Authority i.e., how segregated the area is. However, as previously discussed, the segregation measure (H-Index) captures the *relative* distribution of ethnic groups across a Local Authority. Accordingly, the nature of the measure means it is possible to find higher/lower levels of segregation in areas with higher/lower levels of ethnic diversity (Local Authority segregation and diversity are weakly correlated at *r* = 0.1) (Laurence et al. 2019b). This raises a question: potentially, the stronger impact of engagement on interethnic ties currently observed among participants in more segregated Local Authorities could itself be *conditional* on how much ethnic diversity there is in the Local Authority i.e., participation could exhibit stronger impacts among young people living in Local Authorities that are both more segregated *and* more diverse. For example, while segregation creates barriers to mixing in both higher-diversity and lower-diversity areas, participants from *higher*-diversity segregated areas may experience comparatively greater opportunities for contact when engaging (as higher area diversity translates into more diverse participation-teams), compared to participants in equally segregated areas with lower levels diversity.

To test for this a four-way interaction-term is constructed to explore whether the effect of participation is dependent on both the level of segregation *and* level of diversity in one’s Local Authority, interacting the: pre-/post-test identifier, control/participant identifier, the level of area-segregation variable and the level of area-diversity variable. When fully specified in a model the results showed a positive but non-significant interaction: 1.66 (SE: 1.30 [*CI: −0.86, 4.25*]) (*see* Supplementary-Appendix E for full model results). Therefore, although the relationship is in the predicted direction, any differences in the effects of participation among youth in lower-diversity segregated areas and higher-diversity segregated are not statistically different^10^. Potentially, this may be driven by those dual-processes, outlined above, posited to explain why participation does not exert different impacts among youth from more and less ethnically diverse Local Authorities. These same dual-processes may delimit the size of differences in the effects of participation between *segregated* areas with lower and higher levels of diversity (especially as the data contains no homogeneous Local Authorities; all have a non-trivial level of diversity and thus some opportunities for interethnic contact through participation). However, it could also be driven by insufficient power to robustly test for these differences. The data possess fewer higher-diversity Local Authorities, and when these areas are further disaggregated by levels of segregation, the resulting number of areas in those brackets could inhibit robust tests for differences in the impact of participation. Taken together, while the coefficient is in the expected direction, limitations within the data may restrict fuller testing of this relationship.

#### Robustness testing and ancillary analysis

Several other tests and analyses warrant discussion. A series of DiDiD-models were run which tested whether participation exerted different effects according to young people’s socio-demographics: sex, ethnicity, free school meal status, age, region of residence, the level of Local Authority disadvantage, and whether a respondent is in full-time education. Weakly (*p* < .1) significant relationships demonstrated somewhat stronger effects among youth from more disadvantaged areas and youth not in education. Models were also run testing whether the stronger impact of participation among segregated youth is itself somewhat stronger among different socio-demographic groups. The results found no significant differences. Although, some evidence suggested a somewhat stronger impact among ethnic minorities in segregated areas; however, the relationship was not significant and small ethnic subsamples preclude a fuller test.

A further test focused on an alternative way of conceptualizing contextual-level segregation and diversity, using ethnic-specific relative measures of segregation and diversity for each ethnic group in the sample i.e., based on a young person’s ethnicity, each individual received a relative score of: (a) how segregated their group is in the Local Authority they reside in (the Index of Dissimilarity between *their* ethnic group and all other ethnic groups); and (b) the size of their ethnic out-group in their Local Authority. For example, for Asian participants in the data, their scores would be the Asian/non-Asian Index of Dissimilarity in the Local Authority, and the percent non-Asian in the Local Authority. Similar scores were calculated for White, Black, Mixed and Other groups. When these measures were applied substantively similar findings were returned as previously shown: those participants whose own group was more segregated from others in a Local Authority experienced a stronger positive impact of participation (*see* Supplementary-Appendix D).

## Discussion

Interethnic ties are a key driver of positive interethnic attitudes, especially among adolescents. However, several obstacles exist to developing such ties among young people in society. Youth engagement could offer a key site for fostering greater interethnic connectivity; not only through fostering more optimal contact but, when engagement activities are organized outside of schools, and across neighborhoods, sites of engagement may be able to act as “Associational Bridges”, providing greater opportunities to build ties in more segregated environments. Current studies in the field contain several methodological shortcomings, while no research has systematically tested whether youth engagement can overcome structural barriers to contact. The aim of this study was to overcome these limitations to robustly explore what role youth engagement can play in fostering positive interethnic contact among young people.

Taking advantage of a unique data opportunity in the form of a large-scale, nationally-implemented youth engagement scheme, the findings demonstrated that discrete periods of youth engagement increased young people’s affective interethnic ties, which remained present at least 4–6 months after participation ended. This impact depended to some extent on how segregated the areas were in which young people lived, with participation exerting a much stronger impact on interethnic ties among youth from more segregated areas. However, participation exerted no difference in effects between young people from more or less diverse areas, who both experienced similar gains in contact. Some evidence suggested the stronger impact of participation among youth in more segregated areas was itself even greater when the segregated area was also more diverse. However, the differences observed were not significant, reflecting either a lack of substantive differences or limitations within the data. Further research is required before confirming this finding.

These results partly support the stated hypotheses. As suggested, the nature of the National Citizen Service scheme is likely to produce optimal outgroup contact, with strong “acquaintanceship potential”, leading to greater affective interethnic tie formation (H1). The structure of the scheme should also make it particularly effective for fostering interethnic ties among those from more segregated areas (H2). In theory, youth in segregated areas would be brought together with out-group members from the wider Local Authority with whom they normally have fewer opportunities to mix. The findings support this idea. Young people from integrated areas joined and left the scheme with the same, relatively high level of interethnic ties. Young people from more residentially segregated areas joined the scheme with fewer ties. However, they experienced a much stronger positive effect of participation, and, post-participation, reported similar levels of interethnic ties as their peers from integrated areas.

The study also hypothesized that the scheme’s design should mean participants from more diverse areas experienced a bigger increase in interethnic ties relative to their peers from less diverse areas (H3), given their co-participants were likely more diverse. However, participation had the same effect on youth from more and less diverse Local Authorities. As suggested, one explanation is that while participants from high-diversity areas may have had greater opportunities for mixing on the scheme, they were also more likely to join the scheme already reporting more interethnic ties (which is observed: the number of pre-participation ties was significantly higher among youth from diverse areas). This may limit how much additional scope there was to increase their interethnic ties during participation. Concurrently, while participants in low-diversity areas joined the scheme with fewer interethnic ties, they were also likely to have fewer opportunities to form ties through participation, given low area diversity translates to fewer outgroup co-participants. The result of these two processes in more and less diverse areas may result in no significant difference in effects between them.

These findings make several key contributions to the current literature. The study supports previous evidence that engagement can foster positive youth contact. Interestingly, broadly similar patterns are observed across different socio-demographic groups (with some slightly stronger-effects observed among disadvantaged groups). It also performs the first systematic demonstration that sites of participation can act as “Associational Bridges”, helping overcome structural barriers to contact; particularly residential segregation. Crucially, the findings also show that the interethnic ties persist even after young people are no longer actively involved. Observing this among youth from segregated areas is especially important, given concerns that sustaining contact post-participation could be more difficult for this group (McKeown and Taylor 2017). Through applying a quasi-experimental, intervention approach, confidence that these findings are causal is greater, while the large-scale, nationally-implemented nature of the scheme provides greater scope to generalize findings to society in general.

The results also suggest some caution be taken towards previous cross-sectional work showing participation in more diverse schools, or in more diverse activities, necessarily leads to even greater contact. As observed, young people in more diverse environments already exhibited higher rates of interethnic ties *prior* to engaging. This might be the case for the aforementioned cross-sectional studies: people participating in more diverse environments may already have had higher contact prior to engaging, driving this association. While the ethnic composition of one’s co-participants likely matters for contact, attention needs to be paid to levels of contact prior to participating to more rigorously test the role of co-participant diversity.

Lastly, this study sheds important light on how the design of engagement schemes may affect their ability to overcome structural barriers to contact in society. In the case of the National Citizen Service scheme, participants engage with others from the same Local Authority, and the scheme aims to make the ethnic composition of participants in any Local Authority representative of the wider youth Local Authority population. This design likely leads to particularly positive effects for youth in segregated areas. In turn, this helps to substantially reduce the pre-participation gap in interethnic ties between youth from more and less segregated areas. However, this design likely limits the scheme’s effectiveness for overcoming other structural barriers to contact; especially for closing pre-participation gaps in contact between those from more and less diverse areas (National Audit Office 2017). Although, reassuringly, engagement does not widen these gaps, which could have occurred had engagement led to greater increases in interethnic ties among youth from more diverse areas.

Notwithstanding these insights, this study has limitations. Firstly, using a single measure of interethnic ties limits insights into the processes linking participation to tie formation. The model applied in this study is that participation increases opportunities for contact, the contact experienced through participation is more likely to fulfill the conditions of optimal contact (including “acquaintanceship potential”), which, in turn, leads to affective interethnic ties. Yet, the current study cannot directly measure the intermediary contact stages, such as whether participants experience an increase in their frequency of out-group contact, or the quality of that contact. Evidence for the intermediary stages is instead inferred from the finding that participation generates interethnic ties. One potential piece of evidence supporting the posited model comes from the observation that only participants who ‘enjoyed’ their involvement on the scheme experienced increases in interethnic ties. Assuming that ‘enjoyment’ may operate as a proxy for the quality of participants’ contact during engagement, this could suggest that participation only generates affective interethnic ties when young people experienced more optimal, positive contact with co-participants i.e., for some young people, participation increased contact but if this contact was not experienced positively then inter-ethnic ties did not form. Future research which directly taps both the amount and quality of contact occurring during participation, and then links this to post-participation affective interethnic ties, will provide deeper insights into the intermediary processes at work.

Secondly, the data did not contain explicit measures of the ethnic composition of young people’s teams, inferring mixing opportunities based on the ethnic composition of their Local Authorities. While the administrative data shows the link between team diversity and Local Authority diversity is strong the study cannot explicitly test how team ethnic composition relates to contact. A third limitation involves threats to internal-validity. As selection on to the scheme was not random, differences between participants/controls may lead to different pre-participation trends in ties, which could be driving the engagement-effect. Using an expression of interest control-group, alongside the subsequent PSM-approach, should strengthen the parallel-trends assumption. Tests also showed that not all those who selected on to the scheme saw an increase in ties; instead, their participatory-experience mattered. Furthermore, the observed change in ties among youth from segregated communities in particular provides greater confidence there is a participation-effect: the size of the change, over a 4–6 months period, among young people whose social environment provides fewer contact-opportunities, is unlikely to represent a secular trend in contact among this group.

Threats also exist to generalizing these findings to the scheme as a whole, stemming from survey non-response/missingness. At least on key socio-demographics the participant sample is representative of the participant population as a whole (*see* Supplementary-Appendix C.1). Weighting and multiple-imputation also aimed to minimize bias from baseline/follow-up non-response. However, if people who did not enjoy the scheme (who are less likely to have expanded their intergroup network from participation) were also less likely to respond to the post-test survey the current findings may be upwardly-biased; although reports show the vast majority of participants have positive experiences on the scheme, minimizing such bias (National Audit Office 2017). Future research aims to reduce the impact of these limitations. However, the strength of causal-evidence derived from this large-scale, quasi-experimental approach still makes a key contribution to supplement the current cross-sectional (and smaller scale quasi-experimental) evidence-base.

Lastly, the current study focused on how engagement can build affective interethnic ties, given their important role for intergroup relations. However, as outlined, one side-effect of positive contact can be reduced motivation among disadvantaged, minority-groups to support collective action towards greater ethnic equality (Saguy et al. 2009; Cakal et al. 2011). One posited means of reaping the benefits of positive contact for intergroup relations without sacrificing support for wider social change may be through fostering contact environments in which group differences, and structural ethnic inequalities, are recognized during the interactions (MacInnis and Hodson 2019). Potentially, youth engagement, especially engagement with a social action/civic component, may be well-placed to cultivate such contact. For example, a study of a US community youth program, which aimed to foster not only positive contact but also an awareness of interpersonal and systematic processes of marginalization, found that not only did participants experience an improvement in intergroup attitudes but also an increased commitment towards social justice (Watkins et al. 2007). Future research into youth engagement will benefit from concurrent analyses of collective action outcomes, to explore how (and when) youth engagement may affect both intergroup attitudes but also commitment to collective action, via processes of contact.

A key question which remains from this study is how far the findings could be generalizable to all young people i.e., would the same findings emerge if a random-sample of young people were selected to participate. As outlined, at least on key demographics, participants are broadly representative of the national youth population, providing greater scope for generalizing to all young people. However, certain groups may still be more likely to select on to the scheme which could also affect how participation impacts their interethnic ties; such as more extroverted, prosocial young people, who are more interested in meeting different people. Such traits might predispose them to react more positively to engagement. Concurrently, individuals who are more averse to meeting new people, or mixing with other groups, for whom participation could have a weaker impact, may simply avoid taking part. Some caution is thus required, as in all non-randomized studies, in generalizing to all young people.

Ultimately, the paper provides practical insights for understanding how to foster interethnic ties among young people. It demonstrates robust evidence that non-school, cross-neighborhood youth engagement opportunities are effective for building such ties. Accordingly, encouraging engagement activities in other settings, such as schools and universities, could also be effective at strengthening intergroup cohesion. Furthermore, through its capacity to act as “Associational Bridges”, youth engagement could function as a social-intervention and be applied to ameliorate tensions in areas where intergroup cohesion may be weakest. However, the findings suggest that maximizing the benefits of youth engagement depends, in part, on the design and recruitment practices of the engagement. Program designs which maximize opportunities for contact among young people who have fewer opportunities in their day-to-day lives (for example, drawing participants from across neighborhoods and schools within segregated areas) may yield particularly beneficial results. Yet, careful attention needs to be paid to the design of schemes, given some designs which maximize benefits in one type of area can limit the effectiveness of participation in others. In the present case, while the *within*-Local Authority design of National Citizen Service leads to clear benefits for those in segregated Local Authorities, it limits opportunities for intergroup contact among those living in more ethnically homogeneous areas. As such, the use of bespoke designs for young people from different communities, such as match-making participants from across more and less diverse Local Authorities, could maximize the benefits of engagement for all young people.

## Conclusion

Limitations exist in the current research exploring the role of youth engagement for fostering positive interethnic contact, including: the predominant use of cross-sectional data; the small-scale nature of quasi-experimental tests when they are conducted; a lack of insight into whether any gains in positive contact from engagement persist *after* engagement has ended; and no systematic tests of whether youth engagement can help overcome structural barriers to positive contact in society. Through a large-scale, quasi-experimental, multi-site field study of a nationally-implemented youth engagement scheme, this study aimed to address these limitations to: more robustly explore the effectiveness of youth engagement for fostering affective interethnic ties; test whether any gains in interethnic ties are maintained post-participation; and examine whether engagement can overcome structural obstacles to such ties, such as residential segregation. The findings demonstrated that discrete periods of youth engagement led to increases in young people’s interethnic ties, and that these gains were evident at least 4–6 months after participation had ended. Furthermore, participation had a stronger positive impact on youth from more segregated environments. Taken together, youth engagement schemes can play a critical role in fostering intergroup cohesion. In particular, when acting as “Associational Bridges”, sites of engagement can reduce inequalities in contact among young people by levelling-up contact among those facing greater structural barriers to mixing; especially among youth in more segregated areas.

These results make an important contribution to the study of the opportunities/obstacles to positive contact among adolescents (Titzmann et al. 2015). Given adolescence is a key period during which outgroup attitudes are formed (Kinder and Sears 1981), the importance of affective interethnic ties for positive outgroup attitudes (Pettigrew 1998), but known processes of increasing ethnic homophily in adolescent networks (Brown 2004), understanding the role engagement can play in cross-group ties during adolescence is critical. These results are especially important because while creating opportunities for intergroup contact is demonstrably important for building ties (Titzmann et al. 2015), much of the research into opportunities for contact tends to focus on schools (Bekhuis et al. 2013) and neighborhoods (Merrilees et al. 2018). This research provides new insights into how sites outside of schools and neighborhoods can foster greater contact; especially when obstacles such as residential segregation can limit the effectiveness of schools/neighborhoods for enabling contact. In sum, given rising concern that interethnic attitudes are becoming increasingly polarized (Lambert et al. 2017), this study shows that youth engagement may be able to play a key role in maintaining social harmony in increasingly ethnically diverse societies.

## Supplementary Information

Supplementary Information

## Appendix

Table 2

**Appendix 1:** Pre-matched and post-matched descriptive statistics for participant and control-group samples

	Treated				Control			
	Mean	SD	N	Range	Mean	SD	N	Range
Civic participation in last 3 months								
*Unmatch*	0.68	0.47	1589	0–1	0.63	0.48	2024	0–1
*Matched*	0.67	0.47	1371	0–1	0.69	0.46	1946	0–1
Informal helping in last 3 months								
*Unmatch*	0.73	0.44	1569	0–1	0.72	0.45	2015	0–1
*Matched*	0.73	0.45	1371	0–1	0.74	0.44	1946	0–1
Not Currently studying for A-levels qualification								
*Unmatch*	0.54	0.50	1589	0–1	0.48	0.50	2034	0–1
*Matched*	0.53	0.50	1371	0–1	0.54	0.50	1946	0–1
Index of social confidence								
*Unmatch*	−0.16	0.88	1585	−3.18 to 1.34	−0.07	0.89	2028	−3.18 to 1.34
*Matched*	−0.15	0.87	1371	−3.18 to 1.34	−0.16	0.92	1946	−3.18 to 1.34
Gen. trust cf. Can not be too careful depends								
*Unmatch*	0.54	0.50	1597	0–1	0.51	0.50	2035	0–1
*Matched*	0.54	0.50	1371	0–1	0.52	0.50	1946	0–1
Most can be trusted								
*Unmatch*	0.23	0.42	1597	0–1	0.23	0.42	2035	0–1
*Matched*	0.23	0.42	1371	0–1	0.23	0.42	1946	0–1
Intergroup ties								
*Unmatch*	2.02	0.83	1594	0–3	1.68	0.90	2033	0–3
*Matched*	1.98	0.83	1371	0–3	2.04	0.89	1946	0–3
Local Authority segregation (H Index)								
*Unmatch*	0.16	0.08	1590	0.05–0.5	0.16	0.06	2041	0.05–0.5
*Matched*	0.16	0.08	1371	0.05–0.5	0.16	0.08	1946	0.05–0.5
Local Authority socio-economic disadvantage								
*Unmatch*	0.27	0.95	1590	−1.87 to 3.16	−0.22	0.85	2041	−1.7 to 2.23
*Matched*	0.21	0.92	1371	−1.87 to 3.16	0.18	0.88	1946	−1.7 to 2.23
Local Authority ethnic diversity								
*Unmatch*	0.6	0.41	1590	0.07–1.36	0.36	0.27	2041	0.07–1.36
*Matched*	0.56	0.39	1371	0.07–1.36	0.55	0.35	1946	0.07–1.36
Free school meals eligible (cf. not)								
*Unmatch*	0.2	0.41	1605	0–1	0.16	0.37	2038	0–1
*Matched*	0.2	0.40	1371	0–1	0.19	0.40	1946	0–1
Female (cf. male)								
*Unmatch*	0.63	0.45	1602	0–1	0.68	0.42	2039	0–1
*Matched*	0.64	0.44	1371	0–1	0.62	0.45	1946	0–1
non-White (cf. white)								
*Unmatch*	0.39	0.49	1608	0–1	0.16	0.37	2040	0–1
*Matched*	0.36	0.48	1371	0–1	0.36	0.48	1946	0–1
Cf. 15–16 years old								
16.5 years old								
*Unmatch*	0.15	0.35	1580	0–1	0.16	0.37	2019	0-1
*Matched*	0.15	0.36	1371	0–1	0.15	0.36	1946	0-1
17 years old								
*Unmatch*	0.09	0.28	1580	0–1	0.08	0.28	2019	0–1
*Matched*	0.09	0.29	1371	0–1	0.08	0.28	1946	0–1
17.5 years old								
*Unmatch*	0.3	0.46	1580	0–1	0.29	0.46	2019	0–1
*Matched*	0.29	0.46	1371	0–1	0.3	0.46	1946	0–1
18 years old								
*Unmatch*	0.08	0.27	1580	0–1	0.06	0.25	2019	0–1
*Matched*	0.08	0.47	1589	0–1	0.07	0.26	1946	0–1
cf. North East/North West/Yorkshire and the Humber								
East Midlands/West Midlands								
*Unmatch*	0.13	0.34	1608	0–1	0.23	0.42	2041	0–1
*Matched*	0.14	0.35	1371	0–1	0.16	0.37	1946	0–1
East of England/London/South East/South								
*Unmatch*	0.48	0.50	1608	0–1	0.47	0.50	2041	0–1
*Matched*	0.46	0.50	1371	0–1	0.43	0.50	1946	0–1

PSM is a Epanechnikov kernel-density matching approach with a bandwidth of 0.06, trimmed at the 5% level

## Footnotes

1 This may be because they chose never to participate or participated later in year/the following year.

2 This window covered all regions to form a representative sample of participants.

3 A chi-squared test demonstrates the relationship between sex and responder/non-responder status was significant (χ² = 78.43, *p* < 0.001): young men were less likely to respond than young women. T-tests also demonstrate that, on average, youth who responded to the follow-up survey were (at baseline) more trusting (*t**=* 4.27, *p* < 0.001) and engaged in more civic engagement (*t* = 4.69, *p* < 0.001) than non-responders. Testing also examined whether participation exerted different effects among these sub-groups of young people but found no significant differences.

4 Distinguishing more groups does not change the findings.

5 The calculation of such (un)eveness measures are constrained to some extent by very small numbers of ethnic groups, which cap the maximum and minimum possible levels of segregation. However, this is less applicable in the present study given the size of ethnic groups used in the calculation of the H-Index.

6 Ascertained from NCS Trust administrative data.

7 Based on a four-item index on how confident respondents feel about different areas of their life (5-point likert scale ‘not at all’ to ‘very confident’). “How do you feel about the following things, even if you have never done them before,‘…Meeting new people’,‘…Having a go at things that are new to me’,‘…Working with other people in a team’,‘…Being the leader of a team’” (Eigen Factor: 2.16; minimum loading: 0.69; alpha coefficient: 0.83).

8 As well as leading to greater levels of interethnic contact in more segregated areas, engagement on NCS in segregated areas could also be leading to greater levels of contact between young people from higher and lower levels of socio-economic status (SES). This could occur if, as in the US case, ethnic minority groups in segregated areas tend to be more disadvantaged than their white, majority-group peers. The higher interethnic contact occurring through engagement in segregated areas would therefore also cross SES lines as well, leading to higher inter-SES contact as well. However, both across the country as a whole, and within the data in use, whites and non-whites in segregated areas tend to exhibit, on average, similar disadvantage. In other words, while participation in segregated areas does generate greater inter-ethnic contact, the ties are not, on average, any more/less likely to be inter-SES ties.

9 Derived from Model 5 (Table 1). The least diverse areas have an Entropy score of .15 and the most diverse areas have an Entropy score of 1.35.

10 Another test involved restricting the sample to Whites only, given the only low-diversity areas in the data are those containing high proportions of Whites. However, again, the interaction-term was not significant.
