# Supplementary material for: Youth Engagement, Positive Interethnic Contact, and ‘Associational Bridges’: A Quasi-Experimental Investigation of a UK National Youth Engagement Scheme
Source: J Youth Adolesc. 2019 Jun 7;48(7):1264–80. doi: 10.1007/s10964-019-01042-x (PMC6598961; doi:10.1007/s10964-019-01042-x)
Supplement: Supplementary file 1 — Supplementary Information [file 10964_2019_1042_MOESM1_ESM.docx]

**Supplementary Appendix A – Propensity Score Matching Diagnostics**

**Supplementary-appendix A.1 - Comparison of different matching strategy diagnostics**

| *Matching type* | *Off common-support* | *Mean bias (%)* | *Median bias (%)* | *Rubins' B (%)* | *Rubins' R* | *% of concern* |
| --- | --- | --- | --- | --- | --- | --- |
|  |  |  |  |  |  |  |
| Original sample | 0 | 17.3 | 9.7 | 93.4* | 1.63 | 16 |
| Nearest neighbour (1:1) |  |  |  |  |  |  |
| *No CL; No Rep; no CS* | 0 | 11.8 | 5.3 | 64.3* | 2.44* | 8 |
| *No CL; Without Rep* | 8 | 11.5 | 5.2 | 62.9* | 2.39* | 8 |
| *No CL; With Rep* | 8 | 4.7 | 4.4 | 23.1 | 0.91 | 4 |
| *CL (.08); No Rep*^Ψ^ | 382 | 2.8 | 1.5 | 18.3 | 1.23 | 0 |
| *CL (.08); With Rep* | 8 | 4.7 | 4.4 | 23.1 | 0.91 | 4 |
| *CL (.08); With Rep; trim 5%* | 72 | 4.9 | 4.5 | 22.7 | 0.95 | 4 |
| Nearest neighbour (1:3) |  |  |  |  |  |  |
| *CL (.08); With Rep* | 8 | 5.1 | 4.3 | 24.8 | 0.91 | 0 |
| *CL (.08); With Rep; trim 5%* | 72 | 4.3 | 3.7 | 21.3 | 0.96 | 0 |
| Radius |  |  |  |  |  |  |
| *No CL* | 8 | 17 | 9.5 | 92.1* | 1.35 | 16 |
| *CL (.08)* | 8 | 3.6 | 2.6 | 18.8 | 1.46 | 0 |
| *CL (.08); trim 5%*^Ψ^ | 72 | 2.9 | 2.5 | 15.9 | 1.49 | 0 |
| Kernel |  |  |  |  |  |  |
| *Epanechnikov; Bw (.06)* | 8 | 3.5 | 2.9 | 18.7 | 1.11 | 0 |
| ***Epanechnikov; Bw (.06); trim 5%*^Ψ^** | **72** | **2.8** | **2.5** | **15.6** | **1.19** | **0** |
| *Epanechnikov; Bw (.03)* | 8 | 3.8 | 3.9 | 19.9 | 0.94 | 0 |
| *Epanechnikov; Bw (.03); trim 5%* | 72 | 3.2 | 2.6 | 17.1 | 0.99 | 0 |
| *Gaussian; Bw (.06)* | 8 | 4.7 | 4.4 | 23.1 | 0.91 | 4 |
| *Biweight* | 8 | 4.7 | 4.4 | 23.1 | 0.91 | 4 |

*Notes*: CL = caliper; Rep = replacement; no CS = no common support; Bw = bandwidth; * value outside of suggested range for quality matches (Rubin’s B should be <25 and Rubin’s R between 0.5 and 2) (Rosenbaum and Rubin 1985; Rubin 2001); Ψ signifies strategy under consideration; emboldened strategy selected for analysis. Case order is randomised for nearest-neighbour ‘greedy’ matching. Kernel bandwidths are set to .03 and .06 (Garrido, Kelley et al. 2014). Nearest neighbour/radius calipers are set to 0.25 standard deviations of the logit transformation of the propensity score (Rosenbaum and Rubin 1985).

**Supplementary-appendix A.2 – Covariate balance at pre-participation stage between participants and control group members, before/after matching**

|  |  | Treated | Control | %bias |
| --- | --- | --- | --- | --- |
|  |  |  |  |  |
| Civic participation in last 3 months | Unmatched | 0.68 | 0.63 | 10.5 |
|  | Matched | 0.67 | 0.69 | -3.1 |
|  |  |  |  |  |
| Informal helping in last 3 months | Unmatched | 0.73 | 0.72 | 1.6 |
|  | Matched | 0.73 | 0.74 | -2.3 |
|  |  |  |  |  |
| Not Currently studying for A-levels qualification | Unmatched | 0.54 | 0.48 | 12.6 |
|  | Matched | 0.53 | 0.54 | -1.6 |
|  |  |  |  |  |
| Index of social confidence | Unmatched | -0.16 | -0.07 | -9.7 |
|  | Matched | -0.15 | -0.16 | 1.3 |
| Generalized trust cf. Can't be too careful |  |  |  |  |
| Depends | Unmatched | 0.54 | 0.51 | 6.4 |
|  | Matched | 0.54 | 0.52 | 2.5 |
|  |  |  |  |  |
| Most can be trusted | Unmatched | 0.23 | 0.23 | 0.9 |
|  | Matched | 0.23 | 0.23 | 0.2 |
|  |  |  |  |  |
| Intergroup ties | Unmatched | 2.02 | 1.68 | 39.2 |
|  | Matched | 1.98 | 2.04 | -6.1 |
|  |  |  |  |  |
| Local Authority Segregation (H Index) | Unmatched | 0.16 | 0.16 | 3.7 |
|  | Matched | 0.16 | 0.16 | -2.8 |
|  |  |  |  |  |
| Local Authority socio-economic disadvantage | Unmatched | 0.27 | -0.22 | 54.6 |
|  | Matched | 0.21 | 0.18 | 2.9 |
|  |  |  |  |  |
| Local Authority ethnic diversity (entropy) | Unmatched | 0.6 | 0.36 | 68.2 |
|  | Matched | 0.56 | 0.55 | 5.2 |
|  |  |  |  |  |
| Free school meals eligible (cf. not) | Unmatched | 0.2 | 0.16 | 11.6 |
|  | Matched | 0.2 | 0.19 | 0.7 |
|  |  |  |  |  |
| Female (cf. male) | Unmatched | 0.63 | 0.68 | -12.2 |
|  | Matched | 0.64 | 0.61 | 6.8 |
|  |  |  |  |  |
| non-White (cf. white) | Unmatched | 0.39 | 0.16 | 54.3 |
|  | Matched | 0.36 | 0.36 | -0.3 |
| Cf. 15-16 years old |  |  |  |  |
| 16.5 years old | Unmatched | 0.15 | 0.16 | -3.1 |
|  | Matched | 0.15 | 0.15 | -0.4 |
|  |  |  |  |  |
| 17 years old | Unmatched | 0.09 | 0.08 | 2.1 |
|  | Matched | 0.09 | 0.08 | 2.8 |
|  |  |  |  |  |
| 17.5 years old | Unmatched | 0.3 | 0.29 | 1 |
|  | Matched | 0.29 | 0.3 | -0.7 |
|  |  |  |  |  |
| 18 years old | Unmatched | 0.08 | 0.06 | 6.7 |
|  | Matched | 0.08 | 0.07 | 1 |
| cf. North East/North West/Yorkshire and the Humber |  |  |  |  |
| East Midlands/West Midlands | Unmatched | 0.13 | 0.23 | -26.7 |
|  | Matched | 0.14 | 0.16 | -6.6 |
|  |  |  |  |  |
| East of England/London/South East/South | Unmatched | 0.48 | 0.47 | 3.2 |
|  | Matched | 0.46 | 0.43 | 5 |

**Supplementary-appendix A.3 – Density graph before/after matching**

**Supplementary-appendix A.4 – N of young people on/off common support**

**Supplementary Appendix B – Testing assumptions for valid inference in a Difference-in-Difference approach**

One method of testing the parallel trends assumption is by exploring internal DiD scores among the participant group to examine whether those participants who took part on the scheme but did not report gaining anything from participating experience no impact on their intergroup ties. In other words, we make the assumption that participants who did not find the experience enjoyable will have experienced little impact of the scheme on their out-group ties i.e., it is as if they had not participated. This could help us overcome issues of selection on to the scheme jeopardizing the parallel trends assumption i.e., that young people who joined the scheme had different trends in out-group tie formation before participating: that their levels of contact were already somewhat increasing while the level of ties among non-participants was stable. Therefore, it is these different pre-participation trends accounting for any apparent impact of participation. If we observe that young people who selected on to the scheme but who did not take-away anything from the experience exhibit no impact of participation relative to non-participants we may be more confident that participants did not simply have increasing rates of inter-group contact relative to the controls.

To test this we create binary measures of whether participants reported finding the scheme enjoyable using the following question: ‘Thinking about your National Citizen Service experience overall…On a scale from 0-10, where 0 is not at all enjoyable and 10 is completely enjoyable, how enjoyable did you find this experience?’ The majority of young people reported their experiences as ‘completely enjoyable’ (42%), and 81% reported 8 or above in terms of how enjoyable their experiences of the scheme were. Given this, we need to create broad categories of how enjoyable young people found the scheme to produce a sufficient n for reliable estimates. We therefore divide young people into two categories of experiences: the experience was less enjoyable (rated 0 to 7) and more enjoyable (8 to 10)^[[1]](#endnote-1)^. Using this classification, we divide our participant sample into the two-groups. However, other differences may remain between young people who got ‘less’ and ‘more’ from the scheme which could still affect their trends in intergroup ties. We therefore re-perform the kernel-density PSM approach previously applied but match participants who got ‘less’ from the scheme with suitable control group members and participants who got ‘more’ from the scheme with suitable control group members. Thus, we are minimizing differences in characteristics between the participant and control samples.

Model 1 (**Supplementary-appendix B.1**) shows the results of the matched DiD analysis for the impact of participation on the intergroup ties of young people reporting that their experience was ‘more enjoyable’ (8-10) compared to a matched sample of controls. As previously observed, the DiD term is significant and positive. In fact, it is stronger than we had previously observed among all participants. Model 2 shows the results of the matched DiD analysis for the impact of participation on the intergroup ties of young people who reported their experience as being ‘less enjoyable’ (0-7) compared to a matched sample of controls. We observe that, for this group, the DiD term is small and non-significant, suggesting participation had no impact on their intergroup ties.

There are two key take-aways from this. Firstly, how ‘enjoyable’ a participant found their experience is associated with the strength of the impact of participating. Young people who found the scheme ‘more ‘enjoyable’ report a more positive impact of participating on their intergroup ties. Importantly, this impact is stronger than the positive effect observed among all participants in the main analysis. The second key take-away is that the group who found it ‘less enjoyable’ (who in theory did not derive much value from the scheme) experienced no increase in inter-group contact relative to a matched control group. In other words, despite selecting on to the scheme, those individuals who did not gain anything from participation saw a trend in intergroup ties trend indistinguishable from that of the non-participant group. This strengthens our confidence in the parallel trends assumption.

**Supplementary-appendix B.1**

|  | Model 1 | Model 2 |
| --- | --- | --- |
| Outcome | Interethnic Ties | Interethnic Ties |
| Experienced gained | More enjoyable | Less enjoyable |
|  |  |  |
| cf. Pre-test |  |  |
| Post-test | 0.018 | 0.0132 |
|  | (0.032) | (0.037) |
| cf. Control-group |  |  |
| Participants | 0.053 | 0.055 |
|  | (0.043) | (0.062) |
| Post-test * Participants (DiD) | 0.233*** | -0.003 |
|  | (0.038) | (0.063) |
|  |  |  |
| Constant | 1.908*** | 1.854*** |
|  | (0.035) | (0.043) |
| N | 6075 | 4438 |

Notes: kernel-density (Epanechnikov) propensity-score weighted; bootstrapped standard errors in parentheses (1000 reps);

* p<0.05 ** p<0.01 *** p<0.001 (two-tailed tests)

**Supplementary Appendix C - Tests of External Validity: non-response and missingness**

As discussed, the loss of cases from non-response, both to the pre-test survey and between pre-/post-tests surveys, may bias the external validity of our models. Using NCS administrative data we can explore differences between the full pre-test sample collected^[[2]](#endnote-2)^ and the composition of the participant population that took part during the evaluation period, across key demographics: sex, ethnicity, free school meal status, and region (*see* **Supplementary-appendix C.1**). At least on the available demographics, the pre-test sample and evaluation-period population are highly similar, increasing our confidence that non-response at pre-test is unlikely to significantly bias our findings.

**Supplementary-appendix C.1 – Demographic composition of pre-test sample and evaluation-period population**

|  | Evaluation-period Population | Pre-test Sample |
| --- | --- | --- |
|  | % | % |
| *Sex* |  |  |
| - Male | 39.76 | 39.65 |
| - Female | 60.24 | 60.35 |
| *Ethnicity* |  |  |
| - White | 61.82 | 59.27 |
| - Black | 10.11 | 10.84 |
| - Asian | 19.99 | 20.61 |
| - Mixed | 6.36 | 6.87 |
| - Other | 1.72 | 2.41 |
| *Free School Meal Status* |  |  |
| - Yes | 19.15 | 20.72 |
| - No | 80.85 | 79.28 |
| *Region* |  |  |
| - East Midlands | 7.51 | 6.68 |
| - East of England | 8.19 | 7.25 |
| - London | 24.04 | 26.41 |
| - North East | 6.48 | 8.29 |
| - North West | 17.74 | 18.82 |
| - South East | 10.33 | 12.02 |
| - South West | 3.86 | 1.02 |
| - West Midlands | 9.82 | 6.84 |
| - Yorkshire and The Humber | 12.03 | 12.67 |

*Notes:* evaluation-period population includes those enrolled on the full 4-week program (not the test-program)

A much larger risk to external validity is non-response to the post-test survey. We can explore the drivers of non-response to the post-test survey using the full range of variables applied in this study, given we have full pre-test survey data. **Supplementary-appendix C.2** shows the results of a logistic regression predicting non-response to the post-test survey. Model 1 shows these results for participants. Model 2 shows these results for controls.

**Supplementary-appendix C.2 – Predictors of non-response among participants and controls**

|  | Model 1 | Model 2 |
| --- | --- | --- |
| Dependent variable | Non-response at wave 2 | Non-response at wave 2 |
| Sample | Participant | Control |
|  |  |  |
| Index of social confidence | 0.004 | 0.105** |
|  | (0.039) | (0.035) |
| Local Authority Segregation (H Index) | -0.009 | 0.149 |
|  | (0.535) | (0.651) |
| Local Authority ethnic diversity (entropy) | 0.199 | -0.256 |
|  | (0.144) | (0.164) |
| cf. Studying for A-levels |  |  |
| Not Currently studying for A-levels qualification | 0.070 | 0.010 |
|  | (0.065) | (0.060) |
| Local Authority socio-economic disadvantage | 0.054 | 0.015 |
|  | (0.046) | (0.048) |
| Cf. 15-16 years old |  |  |
| 16.5 years old | 0.053 | -0.019 |
|  | (0.098) | (0.089) |
| 17 years old | -0.064 | 0.118 |
|  | (0.121) | (0.113) |
| 17.5 years old | -0.033 | 0.027 |
|  | (0.079) | (0.072) |
| 18 years old | 0.032 | 0.426*** |
|  | (0.123) | (0.120) |
| cf. North East/North West/Yorkshire and the Humber |  |  |
| East Midlands/West Midlands | -0.054 | 0.033 |
|  | (0.109) | (0.093) |
| East of England/London/South East/South | -0.013 | -0.010 |
|  | (0.119) | (0.100) |
| Generalized trust cf. Can't be too careful |  |  |
| Depends | -0.188* | -0.196** |
|  | (0.079) | (0.070) |
| Most can be trusted | -0.270** | -0.327*** |
|  | (0.095) | (0.086) |
| cf. Male |  |  |
| Female | -0.459*** | -0.385*** |
|  | (0.072) | (0.069) |
| cf. Not FSM eligible |  |  |
| Free school meals eligible | 0.190* | 0.161* |
|  | (0.079) | (0.081) |
| cf. White |  |  |
| non-White | 0.117 | -0.121 |
|  | (0.085) | (0.094) |
|  |  |  |
| Civic participation in last 3 months | -0.209** | -0.174** |
|  | (0.070) | (0.063) |
| Informal helping in last 3 months | 0.141 | 0.089 |
|  | (0.075) | (0.069) |
| Intergroup ties | -0.016 | 0.061 |
|  | (0.041) | (0.035) |
|  |  |  |
| Constant | 1.078*** | 0.732*** |
|  | (0.201) | (0.186) |
| N | 4610 | 4828 |

Notes: * p<0.05 ** p<0.01 *** p<0.001 (two-tailed tests)

These analyses demonstrate significant differences on observable characteristics exist between the composition of our pre-test and post-test survey. Key predictors of non-response include: being male, exhibiting lower generalized trust, and reporting less frequent civic engagement. Reassuringly, on the whole, predictors (and non-predictors) of non-response are similar between participant and control samples. This strengthens confidence in the similarity of the groups. As a note, we create separate non-response weights for our participant and control group samples.

Such attrition may bias the external validity of our analyses. We attempt to account for this by experimenting with creating and applying weights to adjust our post-test sample of participants to be more representative of the participant population during the evaluation-period. Firstly, we apply raking techniques to create a probability weight, based on the population composition of participants during the evaluation-period (*see* **Supplementary-appendix C.1**) to adjust the sample to reflect the demographic composition of the full evaluation-period population (Gelman and Carlin 2002). Secondly, we apply inverse-probability weighting techniques to account for pre-test/post-test attrition, although it remains important to note that the validity of inverse probability weighting is premised on the assumption that attrition is attributable to observables, which is difficult to validate (Weuve et al. 2012). The raked weight, capturing pre-test non-response, will be combined with the inverse-probability weights to form a single weight and trimmed below the 2^nd^ percentile and above the 98^th^ percentile (Himelein 2014). Although such trimming can introduce bias it also can improve the efficiency of our estimates.

As discussed, weights can be used to adjust samples for representativeness within a DiD framework (e.g. Ding and Lehrer 2010). However, applying weights in a PSM-DiD framework is a relatively new area (DuGoff, Schuler and Stuart 2014, Ridgeway et al. 2015). DuGoff, Schuler et al. (2014) discuss options which allow us to get closer to estimating the population ATT (PATT), which is the estimand for the survey’s target population, accounting for the sampling issues, from the unweighted analysis which essentially provides the SATT (Sample Average Treatment Effect amongst the Treated) (Ridgeway et al. 2015). DuGoff, Schuler et al. (2014) suggest the following approach to integrating weights in PSM approaches. Firstly, they suggest including the weight as a covariate within the propensity-score estimation on the assumption that the weight contains information on the probability of responding to the survey which itself may pick up other unobserved characteristics related to both treatment and outcome (DuGoff, Schuler and Stuart 2014). This may help satisfy the assumption of un-confounded treatment assignment. Secondly, it is suggested that the regression analysis, calculating the DiD among the matched-sample, should itself be weighted to allow for inferences regarding the PATT (not SATT only); that is, the impact of participation on all participants during the evaluation-period (and, given these participants are themselves representative of all summer participants, by association the impact of participation for the 2015 summer cohort of participants).

How can we apply these techniques in our own study? It is important to note that for our ‘participant sample’ we have two sets of weights: a raked-weight to account for sample representativeness due to non-response at wave-1; and an IPW to account for non-response at wave-2. However, for our control group we only have one weight: the IPW to account for non-response at wave-2, given we do not have information on the demographic composition of the total population of young people in the control group from which sample was drawn. Therefore, we cannot create raked-weights to adjust for this. This has implications for how we can take the suggestions made by DuGoff, Schuler et al. (2014). To include the weights as a covariate within the propensity-score model they need to be comparable between treatment and control groups. Therefore, we can only attempt this stage if we restrict ourselves to focusing on the IPW for both groups (and excluding the role of the raked-weights). This may not be problematic, given, as we have seen, the demographic similarity between the pre-test sample of participants and the total population of participants during the evaluation period. In part, this stems from the high response rate among participants to the baseline survey (85%). Furthermore, it is unclear what the ‘total population’ of the control group necessarily is i.e., what would we be making it representative of, *per se.* However, one test we can conduct is weighting the control-group up to the population of the participants. This is based on the assumption that we want to make the groups as comparable as possible.

An alternative approach is to exclude any weights from the propensity score calculation and just follow the second, and more important, suggestion of DuGoff, Schuler et al. (2014) to weight the final regression models calculating the DiD score. This will allow us to take a number of approaches. We could create a single weight for our participant group, combining the raked-weights and IPW. This would account for non-response at both the pre-test and post-test stage for participants. We can then simply use the IPW for the control-group, to account for post-test non-response. Alternatively, we could simply weight both the participant and control groups by their IPW alone. Importantly, as our PSM approach involved kernel-density weighting (which requires analyses to apply to the kernel weight) the kernel-weights can be combined with the non-response weights in the regression analysis (DuGoff, Schuler and Stuart 2014).

We will therefore run a number of different specifications to examine how accounting for various sources of bias, in both our participant and control group samples, may impact of findings. This will include:

1. Propensity score stage: no weights included for participant and control samples. Regression stage: IPW weights included for participant and control samples.
2. Propensity score stage: IPW weights included for participant and control samples. Regression stage: IPW weights included for participant and control samples.
3. Propensity score stage: no weights included for participant and control samples. Regression stage: IPW and raked weights included for participant sample only.
4. Propensity score stage: no weights included for participant and control samples. Regression stage: IPW and raked weights included for participant sample and IPW included for control sample.
5. Propensity score stage: no weights included for participant and control samples. Regression stage: IPW and raked weights included for participant and control samples.

**Supplementary-appendix C.3** shows the results of the weighted analysis, applying the approaches outlined above. As discussed, the IPW weights were created using all variables available. Separate IPW weights were created for participant and control groups to account for any differences in drivers of attrition between the two groups. We replicate our key models (that is, the overall impact of participation on life satisfaction (Model 1, **Table 1**) and the moderated impact of participation by community disadvantage (Model 3, **Table 1**)) but applying different weighting strategies. Models 1-2 (**Supplementary-appendix C.3**) weight the models using IPW for both participant and control groups. The findings are consistent with our previous analysis. Models 3-4 includes the IPW, for both participant and controls, in the PSM stage and then weight the final models using the same weights. Matching is conducted using the same covariates and kernel-density approach but including the weights in the calculation of the propensity score. Both the overall impact and DiDiD term remain consistent with previous findings (Models 3 and 4). Models 5-6 weight the models using the combined raked-weight and IPW for the participant sample only (controls are set to the kernel density weight alone). The findings are consistent with our previous analysis. Models 7-8 weight the models using the combined raked-weight and IPW for the participant sample and the IPW for the control-group (again, combined with the kernel density weights). The findings are consistent with our previous analysis. Models 9 and 10 weight the models using the combined raked-weight and IPW for the participant *and* control sample (where the control sample is weighted to represent the participant population). On the whole, the various weighted analyses show a high degree of consistency with our key findings, which is especially important given the tendency of weights to inflate standard errors. This suggests the loss of cases does not significantly bias our findings, insofar as the available observable characteristics are effective at accounting for drivers of attrition.

**Supplementary-appendix C.3 - Tests for external validity bias: weighting**

|  | Model 1 | Model 2 | Model 3 | Model 4 | Model 5 | Model 6 | Model 7 | Model 8 |
| --- | --- | --- | --- | --- | --- | --- | --- | --- |
| Outcome | Interethnic Ties | Interethnic Ties | Interethnic Ties | Interethnic Ties | Interethnic Ties | Interethnic Ties | Interethnic Ties | Interethnic Ties |
|  |  |  |  |  |  |  |  |  |
|  |  |  |  |  |  |  |  |  |
| cf. Pre-test |  |  |  |  |  |  |  |  |
| Post-test | 0.046 | 0.182* | 0.038 | 0.168* | 0.046 | 0.182* | -0.007 | 0.124 |
|  | (0.033) | (0.073) | (0.036) | (0.075) | (0.033) | (0.073) | (0.065) | (0.149) |
| cf. Control-group |  |  |  |  |  |  |  |  |
| Participants | 0.137*** | 0.180+ | 0.103* | 0.144 | 0.134*** | 0.166 | 0.094 | 0.118 |
|  | (0.040) | (0.101) | (0.040) | (0.100) | (0.040) | (0.102) | (0.059) | (0.143) |
| Post-test * Participants (DiD) | 0.165*** | 0.007 | 0.178*** | 0.041 | 0.170*** | 0.027 | 0.223** | 0.084 |
|  | (0.038) | (0.087) | (0.041) | (0.092) | (0.039) | (0.089) | (0.068) | (0.157) |
| Area Segregation |  | -1.084** |  | -1.053** |  | -1.083** |  | -0.784 |
|  |  | (0.389) |  | (0.402) |  | (0.393) |  | (0.492) |
| Post-test * Area Segregation |  | -0.418 |  | -0.433 |  | -0.418 |  | -0.797 |
|  |  | (0.414) |  | (0.436) |  | (0.414) |  | (0.636) |
| Participants * Area Segregation |  | 0.161 |  | 0.176 |  | 0.208 |  | -0.135 |
|  |  | (0.545) |  | (0.543) |  | (0.548) |  | (0.668) |
| Post-test * Participants * Area Segregation (DiDiD) |  | 1.151** |  | 1.078* |  | 1.063** |  | 1.440* |
|  |  | (0.394) |  | (0.419) |  | (0.395) |  | (0.597) |
| Area Ethnic Diversity |  | 0.984*** |  | 0.959*** |  | 0.979*** |  | 0.906*** |
|  |  | (0.099) |  | (0.102) |  | (0.099) |  | (0.120) |
| Post-test * Area Ethnic Diversity |  | -0.117 |  | -0.105 |  | -0.117 |  | -0.007 |
|  |  | (0.096) |  | (0.105) |  | (0.096) |  | (0.149) |
| Participants * Ethnic Diversity |  | -0.320** |  | -0.297** |  | -0.307** |  | -0.192 |
|  |  | (0.106) |  | (0.108) |  | (0.108) |  | (0.146) |
| Post-test * Participants * Area Ethnic Diversity (DiDiD) |  | -0.047 |  | -0.067 |  | -0.055 |  | -0.165 |
|  |  | (0.097) |  | (0.107) |  | (0.100) |  | (0.148) |
|  |  |  |  |  |  |  |  |  |
|  |  |  |  |  |  |  |  |  |
| Constant | 1.813*** | 1.592*** | 1.849*** | 1.623*** | 1.812*** | 1.593*** | 1.823*** | 1.602*** |
|  | (0.033) | (0.065) | (0.034) | (0.067) | (0.033) | (0.065) | (0.044) | (0.097) |
|  |  |  |  |  |  |  |  |  |
| N (individuals) | 3311 | 3311 | 3311 | 3311 | 3311 | 3311 | 3311 | 3311 |
| N (Local Authorities) | 302 | 302 | 302 | 302 | 302 | 302 | 302 | 302 |

Notes: kernel-density (Epanechnikov) propensity-score weighted; bootstrapped standard errors in parentheses (1000 reps);

* p<0.05 ** p<0.01 *** p<0.001 (two-tailed tests)

A second threat to the external validity is from missing data within cases among participant/control cases who completed both the pre-test/post-test surveys. Importantly, missing data never exceeds 2% on any one variable and most variables have less than 1% missing data (*see* **Supplementary-appendix C.4**). This is reassuring suggesting respondents did not appear to find any questions particularly difficult or uncomfortable. One way of dealing with such missingness is to apply listwise deletion of cases with missing data. However, while per variable missingness is low, when all cases with at least one piece of missing data are taken into account we would lose n=262 cases across our participant/control groups.

**Supplementary-appendix C.4 - N and % of missing on each variable used in the study**

| Variable | Observations (n) | Missings (n) | % missing |
| --- | --- | --- | --- |
|  |  |  |  |
| Index of social confidence | 7298 | 70 | 0.9592 |
| Not Currently studying for A-levels qualification | 7298 | 52 | 0.7125 |
| Local Authority socio-economic disadvantage | 7298 | 36 | 0.4933 |
| Local Authority ethnic diversity (entropy) | 7298 | 36 | 0.4933 |
| Local Authority Segregation (H Index) | 7298 | 36 | 0.4933 |
| Positive Contact | 7298 | 39 | 0.5344 |
| Age | 7298 | 100 | 1.37 |
| Region | 7298 | 0 | 0 |
| Generalized Trust | 7298 | 33 | 0.4522 |
| Sex | 7298 | 26 | 0.3563 |
| FSM Status | 7298 | 23 | 0.3152 |
| Ethnicity | 7298 | 10 | 0.137 |
| Civic participation in last 3 months | 7298 | 100 | 1.37 |
| Informal helping in last 3 months | 7298 | 119 | 1.631 |

One method of addressing such within-case missingness is through multiple-imputation (MI) using chained-equations. As with weighting, this technique again relies on the assumption that within-case missing data is missing at random (MAR). MI can be combined with PSM to generate propensity scores based on multiple imputated data sets^[[3]](#endnote-3)^ (Lunt 2011). With 11% of cases lost to within-case missing data across all variables we generate 20 imputed datasets, using all covariates and mechanism variables used in our models, as well as using the outcome variable to inform the value imputation but not imputing values for the outcome variable (Graham, Olchowski and Gilreath 2007, Mostafa and Wiggins 2015, Sterne et al. 2009). Imputed values for binary and categorical variables were restricted to actual values available for those variables. Estimates from the analyses were combined according to Rubin’s rules (White, Royston and Wood 2011).

Using the multiple-imputed datasets we re-ran our analysis, including the PSM, on the imputed datasets. We also experimented with re-creating our IPW weights using the fully imputed dataset, to minimize the bias caused by missing data in their initial calculation. These weights are then included in the MI model (as performed in Models 1 and 2 (**Supplementary-appendix C.3**)). **Supplementary-appendix C.5** shows the results of the MI analysis. Models 1 and 2 replicate our key findings using the kernel-density matching procedure applied previously. These results are highly consistent with our key findings. As discussed, the application of MI and weighting to PSM approaches is a relatively new area of analysis. However, it is reassuring that our findings remain consistent and robust with the application of these approaches.

**Supplementary-appendix C.5 – Multiple-imputation**

|  | Model 1 | Model 2 |
| --- | --- | --- |
| Outcome | Interethnic Ties | Interethnic Ties |
|  |  |  |
|  |  |  |
| cf. Pre-test |  |  |
| Post-test | 0.045 | 0.162* |
|  | (0.034) | (0.077) |
| cf. Control-group |  |  |
| Participants | -0.032 | 0.135 |
|  | (0.071) | (0.177) |
| Post-test * Participants (DiD) | 0.173*** | 0.036 |
|  | (0.039) | (0.093) |
| Area Segregation |  | -0.758 |
|  |  | (0.756) |
| Post-test * Area Segregation |  | -0.38 |
|  |  | (0.422) |
| Participants * Area Segregation |  | -0.871 |
|  |  | (0.852) |
| Post-test * Participants * Area Segregation (DiDiD) |  | 1.126** |
|  |  | (0.396) |
| Area Ethnic Diversity |  | 1.07*** |
|  |  | (0.165) |
| Post-test * Area Ethnic Diversity |  | -0.095 |
|  |  | (0.09) |
| Participants * Ethnic Diversity |  | -0.249 |
|  |  | (0.174) |
| Post-test * Participants * Area Ethnic Diversity (DiDiD) |  | -0.076 |
|  |  | (0.093) |
|  |  |  |
|  |  |  |
| Constant | 1.818 | 1.609 |
|  | (0.032) | (0.066) |
| N (observations) | 7204 | 7204 |

Notes: kernel-density (Epanechnikov) propensity-score weighted; bootstrapped standard errors in parentheses (1000 reps);

* p<0.05 ** p<0.01 *** p<0.001 (two-tailed tests)

**Supplementary Appendix D – Control-group and Matching and Model Specifications**

We aim to examine the sensitivity of our findings to the matching method applied, model specifications, and the control-group selected (**Supplementary Appendix D.1**). The first test involves examining the sensitivity to the matching specifications by re-running our key models but using two alternative matching selections that had similarly lower levels of bias (*see* **Supplementary-appendix A.2**): Models 1 and 2 apply a one-to-one matching method (with common support, a caliper at .08, and no replacement); and Models 3 and 4 apply a radius-matching approach (with common support, a caliper at .08, and 5% trim of the propensity score). We observe that the models produce highly similar findings to those reported in the main paper. The second tests apply doubly-robust methods which involves re-running our key models thus far (using the kernel-density matching approach) but also including all the variables applied in the matching-process into the regression models to adjust estimates for the effect of any remaining imbalance from the matching process. These findings also demonstrate consistency with those reported in the main body of the paper (Models 5 and 6). Thirdly, we explore how sensitive our findings are to control-group selection by testing the overall impact of participation using a second control-group. This is drawn from the 2015 Voice of Youth (VoY) survey. The VoY data is a nationally representative sample of 16-18 years olds, conducted over the same pre-test/post-test period as the NCS data, composed of n=662 young people. Due to cost implications, a postal survey was taken, using the National Pupil Database as the sampling frame. The pre-test response rate was 18% and the post-test response rate was 43%. We apply the same kernel-density matching approach as previously applied. The results show substantively similar findings as those reported previously, with a DiD-score of: 0.22 [*CI: 0.11, 0.33*] (Model 7). In other words, even using a different control-group the findings remain robust. Unfortunately, we cannot conduct this test into the moderating impact of segregation as we lack the area identifiers in the VoY data.

This approach can also be used to perform a ‘placebo’ test, whereby a ‘fake’ treatment group are used instead of participants and compared to the control group (Gertler, Martinez et al. 2016). The absence of any impact should again provide evidence in favour of the parallel trends assumption. In our case, using the VoY control group as the ‘fake’ treatment group, we observe that the pre-test/post-test trends for the ‘expression of interest’ control group and the VoY control group are not significantly different from one another.

We also tested the use of ethnic-group specific relative measures of segregation and ethnic diversity instead of the H and Entropy scores (Model 8). Each participant receives a relative score of: (a) how segregated their group is in the Local Authority they reside in (the Index of Dissimilarity between *their* ethnic group and all other ethnic groups); (b) the size of the ethnic out-group in their Local Authority, based on their ethnicity. For example, for Asian participants, this would the Asian/non-Asian Index of Dissimilarity score in the Local Authority, and the percent non-Asian in the Local Authority; for Black participants this would be the Black/non-Black Index of Dissimilarity score for the Local Authority, and the percent non-Black in the Local Authority. Similar scores were calculated for White, Mixed and Other groups as well. Therefore, within any given Local Authority, different ethnic groups would have different relative scores of segregation and diversity. Substantively similar findings are returned as those reported in the main findings.

**Supplementary-appendix D.1 – Modelling, matching and control-group tests**

|  | Model 1 | Model 2 | Model 3 | Model 4 | Model 5 | Model 6 | Model 7 | Model 8 |
| --- | --- | --- | --- | --- | --- | --- | --- | --- |
|  | Interethnic Ties | Interethnic Ties | Interethnic Ties | Interethnic Ties | Interethnic Ties | Interethnic Ties | Interethnic Ties | Interethnic Ties |
| Test | 1-to-1 match | 1-to-1 match | Radius match | Radius match | Doubly robust | Doubly robust | Voy Control-group | Group relative measures |
|  |  |  |  |  |  |  |  |  |
| cf. Pre-test |  |  |  |  |  |  |  |  |
| Post-test | 0.127*** | 0.177** | 0.045 | 0.130* | 0.030 | 0.147* | -0.015 | 0.117 |
|  | (0.026) | (0.062) | (0.027) | (0.059) | (0.032) | (0.070) | (0.054) | (0.079) |
| cf. Control-group |  |  |  |  |  |  |  |  |
| Participants | 0.027 | 0.140 | 0.076* | 0.132 | 0.015 | 0.188+ | 0.136* | 0.161 |
|  | (0.040) | (0.101) | (0.037) | (0.092) | (0.036) | (0.101) | (0.067) | (0.122) |
| Post-test * Participants (DiD) | 0.149*** | 0.012 | 0.182*** | 0.059 | 0.194*** | 0.048 | 0.221*** | -0.009 |
|  | (0.036) | (0.083) | (0.033) | (0.079) | (0.037) | (0.087) | (0.059) | (0.103) |
| Area Segregation |  | -1.167** |  | -1.260*** | -0.388 | -0.780+ |  |  |
|  |  | (0.380) |  | (0.347) | (0.262) | (0.407) |  |  |
| Post-test * Area Segregation |  | -0.190 |  | 0.018 |  | -0.042 |  |  |
|  |  | (0.298) |  | (0.312) |  | (0.345) |  |  |
| Participants * Area Segregation |  | 0.317 |  | 0.404 |  | 0.098 |  |  |
|  |  | (0.551) |  | (0.498) |  | (0.551) |  |  |
| Post-test * Participants * Area Segregation (DiDiD) |  | 0.932* |  | 0.678* |  | 0.690* |  |  |
|  |  | (0.387) |  | (0.346) |  | (0.358) |  |  |
| Area Ethnic Diversity |  | 0.900*** |  | 0.983*** | 0.371*** | 0.716*** |  |  |
|  |  | (0.106) |  | (0.106) | (0.065) | (0.107) |  |  |
| Post-test * Area Ethnic Diversity |  | -0.040 |  | -0.165+ |  | -0.200* |  |  |
|  |  | (0.085) |  | (0.094) |  | (0.090) |  |  |
| Participants * Ethnic Diversity |  | -0.377** |  | -0.383*** |  | -0.409*** |  |  |
|  |  | (0.125) |  | (0.116) |  | (0.113) |  |  |
| Post-test * Participants * Area Ethnic Diversity (DiDiD) |  | -0.058 |  | 0.030 |  | 0.067 |  |  |
|  |  | (0.116) |  | (0.100) |  | (0.097) |  |  |
| Area in-group/out-group Segregation |  |  |  |  |  |  |  | -0.522** |
|  |  |  |  |  |  |  |  | (0.203) |
| Post-test * in-group/out-group segregation |  |  |  |  |  |  |  | -0.056 |
|  |  |  |  |  |  |  |  | (0.202) |
| Participants * in-group/out-group segregation |  |  |  |  |  |  |  | -0.177 |
|  |  |  |  |  |  |  |  | (0.308) |
| Post-test * Participants * in-group/out-group segregation (DiDiD) |  |  |  |  |  |  |  | 0.522* |
|  |  |  |  |  |  |  |  | (0.253) |
| Area % out-group |  |  |  |  |  |  |  | 0.900*** |
|  |  |  |  |  |  |  |  | (0.058) |
| Post-test * Area % out-group |  |  |  |  |  |  |  | -0.192** |
|  |  |  |  |  |  |  |  | (0.061) |
| Participants * Area % out-group |  |  |  |  |  |  |  | -0.212** |
|  |  |  |  |  |  |  |  | (0.080) |
| Post-test * Participants * Area % out-group (DiDiD) |  |  |  |  |  |  |  | -0.004 |
|  |  |  |  |  |  |  |  | (0.088) |
|  |  |  |  |  |  |  |  |  |
| Constant | 1.829*** | 1.645*** | 1.864*** | 1.657*** | 1.655*** | 1.557*** | 1.850*** | 1.850*** |
|  | (0.034) | (0.071) | (0.032) | (0.060) | (0.089) | (0.100) | (0.067) | (0.081) |
| N | 4234 | 4234 | 6622 | 6622 | 6622 | 6622 | 3315 | 6622 |

Notes: bootstrapped standard errors in parentheses (1000 reps);

* p<0.05 ** p<0.01 *** p<0.001 (two-tailed tests)

**Supplementary Appendix E.1 – Tests for conditional impact of participation by segregation *and* diversity**

|  | Model 1 |
| --- | --- |
|  | Interethnic Ties |
|  |  |
| cf. Pre-test |  |
| Post-test | -0.110 |
|  | (0.144) |
| cf. Control-group |  |
| Participants | -0.090 |
|  | (0.190) |
| Post-test * Participants (DiD) | 0.233 |
|  | (0.178) |
| Area Segregation | -2.753** |
|  | (0.841) |
| Post-test * Area Segregation | 1.703 |
|  | (0.886) |
| Participants * Area Segregation | 1.937 |
|  | (1.233) |
| Post-test * Participants * Area Segregation (DiDiD) | -0.550 |
|  | (1.056) |
| Area Ethnic Diversity | 0.675** |
|  | (0.210) |
| Post-test * Area Ethnic Diversity | 0.153 |
|  | (0.204) |
| Participants * Ethnic Diversity | -0.068 |
|  | (0.280) |
| Post-test * Participants * Area Ethnic Diversity (DiDiD) | -0.194 |
|  | (0.229) |
| Area Segregation * Area Diversity | 1.151 |
|  | (1.207) |
| Post-test * Area Segregation * Area Diversity | -1.365 |
|  | (1.144) |
| Participants * Area Segregation * Area Diversity | -1.194 |
|  | (1.736) |
| Post-test * Participants * Area Segregation * Area Diversity | 1.694 |
|  | (1.305) |
|  |  |
| Constant | 2.872*** |
|  | (0.128) |
|  |  |
| N (individuals) | 3311 |
| N (Local Authorities) | 302 |

Notes: bootstrapped standard errors in parentheses (1000 reps);

* p<0.05 ** p<0.01 *** p<0.001 (two-tailed tests)

**REFERENCES:**

Caliendo, Marco and Sabine Kopeinig. 2008. "Some Practical Guidance for the Implementation of Propensity Score Matching." *Journal of Economic Surveys* 22(1):31-72.

Ding, Weili and Steven F. Lehrer. 2010. "Estimating Treatment Effects from Contaminated Multiperiod Education Experiments: The Dynamic Impacts of Class Size Reducations." *The Review of Economics and Statistics* 92(1):31-42.

DuGoff, Eva H., Megan Schuler and Elizabeth A. Stuart. 2014. "Generalizing Observational Study Results: Applying Propensity Score Methods to Complex Surveys." *Health Services Research* 49(1):284-303.

Garrido, Melissa M., Amy S. Kelley, Julia Paris, Katherine Roza, Diane E. Meier, R. Sean Morrison and Melissa D. Aldridge. 2014. "Methods for Constructing and Assessing Propensity Scores." *Health Services Research* 49(5):1701-20.

Gelman, Andrew and John B. Carlin. 2002. "Poststratification and Weighting Adjustments " Pp. 289-302 in *Survey Nonresponse*, edited by R. Groves, D. Dillman, J. Eltinge and R. Little. New York: Wiley.

Graham, John W., Allison E. Olchowski and Tamika D. Gilreath. 2007. "How Many Imputations Are Really Needed? Some Practical Clarifications of Multiple Imputation Theory." *Prevention Science* 8(3):206-13.

Guo, S. and M. W. Fraser. 2009. "Propensity Score Analysis: Statistical Methods and Applications." Thousand Oaks, CA: Sage.

Heckman, James, Hidehiko Ichimura, Jeffrey Smith and Petra Todd. 1998. "Characterizing Selection Bias Using Experimental Data." *Econometrica* 66(5):1017-98.

Himelein, Kristen. 2014. "Weight Calculations for Panel Surveys with Subsampling and Split-Off Tracking." *Statistics and Public Policy* 1(1):40-45.

Lunt, Mark. 2011. "A Guide to Imputing Missing Data with Stata: Revision 1.4."

Mostafa, Tarek and Richard Wiggins. 2015. "The Impact of Attrition and Non-Response in Birth Cohort Studies: A Need to Incorporate Missingness Strategies." *Longitudinal and Life Course Studies* 6(2):131-46.

Ridgeway, Greg, Stephanie Ann Kovalchik, Beth Ann Griffin and Mohammed U. Kabeto. 2015. "Propensity Score Analysis with Survey Weighted Data." *Journal of causal inference* 3(2):237-49.

Rosenbaum, Paul R. and Donald B. Rubin. 1985. "Constructing a Control Group Using Multivariate Matched Sampling Methods That Incorporate the Propensity Score." *The American Statistician* 39(1):33-38.

Sterne, Jonathan A. C., Ian R. White, John B. Carlin, Michael Spratt, Patrick Royston, Michael G. Kenward, Angela M. Wood and James R. Carpenter. 2009. "Multiple Imputation for Missing Data in Epidemiological and Clinical Research: Potential and Pitfalls." *BMJ* 338.

Weuve, Jennifer, Eric J. Tchetgen Tchetgen, M. Maria Glymour, Todd L. Beck, Neelum T. Aggarwal, Robert S. Wilson, Denis A. Evans and Carlos F. Mendes de Leon. 2012. "Accounting for Bias Due to Selective Attrition: The Example of Smoking and Cognitive Decline." *Epidemiology (Cambridge, Mass.)* 23(1):119-28.

White, Ian R., Patrick Royston and Angela M. Wood. 2011. "Multiple Imputation Using Chained Equations: Issues and Guidance for Practice." *Statistics in medicine* 30(4):377-99.

1. Experimenting with narrower definitions of ‘less enjoyable’ (e.g. <7, or <6) produces broadly similar results, although the trend becomes somewhat more negative. However, the substantially reduced n makes estimations less reliable. [↑](#endnote-ref-1)
2. Less than 3.38% of cases in the pre-test full sample had missing data on these key demographic characteristics [↑](#endnote-ref-2)
3. Multiple imputation was undertaken in Stata 14 using the *ice, nscore* and *mim* programs, following Lunt (2011). [↑](#endnote-ref-3)
